# Integrative In Silico Profiling of Major Phytochemicals From a *Sclerocarya birrea–Nauclea latifolia–Piper longum* Mixture: Docking, Molecular Dynamics, ADMET, and Ligand‐Based Target Prediction on Shared Pathways in Type 2 Diabetes Mellitus and Alzheimer's Disease

**DOI:** 10.1002/open.70299

**Published:** 2026-09-15

**Authors:** Jean Philippe Djientcheu Tientcheu, Erman Salih İstifli

**Affiliations:** ^1^ Department of Animal Biology and Physiology University of Yaoundé I Yaoundé Cameroon; ^2^ Faculty of Science and Literature Department of Biology Cukurova University Adana Türkiye

**Keywords:** molecular dynamics, multi‐target phytochemical discovery, SNP‐mixture, type 2 diabetes mellitus (T2DM)–Alzheimer's disease (AD) axis

## Abstract

Type 2 diabetes mellitus (T2DM) and Alzheimer's disease (AD) converge through metabolic dysfunction, inflammation, and impaired neuronal signaling, providing a rationale for multi‐target intervention. This study aimed to identify phytochemicals from a *Sclerocarya birrea–Nauclea latifolia–Piper longum* mixture that may modulate this disease axis. Seven constituents were screened against AChE, BACE1, DPP4, glucokinase, GSK3β, and TACE using molecular docking and MM‐GBSA rescoring. Prioritized complexes underwent triplicate 100‐ns molecular dynamics simulations, post‐MD MM‐GBSA analysis, physicochemical/ADMET profiling, and ligand‐based target prediction. Quercetin pentamethyl ether (QR‐PME) emerged as the principal computational hit: its GSK3β and TACE complexes maintained stable structural and energetic behavior, with mean binding free energy estimates of −30.07 ± 0.06 and −34.24 ± 0.07 kcal/mol, respectively, matching those of the reference inhibitors (−30.05 ± 0.07 and −34.08 ± 0.06 kcal/mol). QR‐PME satisfied Lipinski criteria and showed predicted intestinal absorption, although CYP inhibition liability and low blood–brain barrier permeability remain concerns. ABCG2 was its top predicted target, suggesting an experimentally unresolved link to amyloid‐β transport. These findings generate a testable hypothesis that QR‐PME may engage the metabolic–inflammatory–tau network connecting T2DM and AD, supporting biochemical, cellular, pharmacokinetic, and in vivo validation before its therapeutic applicability can be established.

## Graphical abstract

Which major phytochemical from the Sclerocarya birrea–Nauclea latifolia–Piper longum mixture engages shared targets across the T2DM–AD axis? Integrated docking, triplicate 100‐ns molecular dynamics, post‐MD MM‐GBSA, and ADMET/target prediction prioritize QR‐PME, which forms stable GSK3β and TACE complexes with binding energy estimates comparable to reference inhibitors. The findings define a testable metabolic–inflammatory–tau hypothesis requiring experimental validation.

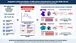

## 1 Introduction

Type 2 diabetes mellitus (T2DM) and Alzheimer's disease (AD) are among the most prevalent chronic disorders worldwide, particularly in older adults [1, 2, 3, 4]. T2DM is characterized by persistent hyperglycemia and metabolic dysfunction [5, 6] and is increasingly associated with a more rapid decline in neurocognitive function, alongside a greater risk of AD [2, 7]. AD, a progressive neurodegenerative disorder, is pathologically characterized by extracellular amyloid‐β deposits (plaques) and intraneuronal neurofibrillary tangles and is further associated with mitochondrial dysfunction, chronic neuroinflammation, cholinergic deficits, and increased oxidative stress [8, 9, 10]. In 2021, diabetes affected over 537 million adults worldwide, and this burden is expected to increase to ~783 million by 2045 [11, 12], whereas dementia currently affects over 55 million people worldwide, 60%–70% of whom have AD [7, 13, 14]. Consistent with the conceptual notion of “*type 3 diabetes,*” both T2DM and AD converge on several pathogenic pathways, notably oxidative stress, insulin resistance, inflammatory signaling, and mitochondrial impairment [2, 7, 15, 16, 17]—which underscores their intertwined pathophysiology and combined public health impact.

Epidemiological studies and meta‐analyses have consistently reported a higher incidence of cognitive impairment and dementia in people with diabetes; part of this association, however, appears to be mediated by cerebrovascular pathways [18, 19, 20, 21]. Against this background, pharmacological management of T2DM and AD still relies largely on conventional drug classes—sulfonylureas, meglitinides, biguanides, gliptins, thiazolidinediones, and metformin for T2DM and rivastigmine, galantamine, tacrine, and donepezil for AD [5, 6, 8, 22]. However, these agents remain insufficient to halt disease progression or to reduce the overall disease burden at the population level, and their use is frequently limited by adverse effects such as gastrointestinal disturbances, hypoglycemia, hepatotoxicity, and increased cardiovascular risk [13, 23, 24, 25].

In T2DM and AD, therapeutic efforts have traditionally focused on well‐established pathways, including insulin and incretin signaling in T2DM and amyloid‐β, tau, and cholinergic neurotransmission in AD [26, 27]. However, accumulating experimental and clinical evidence suggests that these disorders are connected through a broader metabolic–neurodegenerative axis. Chronic hyperglycemia and impaired insulin signaling promote oxidative stress, advanced glycation end‐product formation, vascular dysfunction, and low‐grade inflammation, thereby contributing to cerebral insulin resistance, neuroinflammation, and cognitive decline [28, 29]. Within this interconnected network, glucokinase (GK) activation and dipeptidyl peptidase‐4 (DPP4) inhibition represent upstream interventions that may improve glucose sensing, incretin activity, and systemic glucose homeostasis [5, 25, 29, 30, 31, 32]. These metabolic effects may act synergistically with the direct modulation of downstream AD‐related processes through inhibition of β‐secretase 1 (BACE1), glycogen synthase kinase‐3β (GSK3β), acetylcholinesterase (AChE), and tumor necrosis factor‐α converting enzyme (TACE/ADAM17). Collectively, these targets regulate amyloidogenic processing, tau hyperphosphorylation, cholinergic neurotransmission, and TNF‐α‐mediated neuroinflammatory signaling [7, 8, 22, 33]. Although synthetic modulators of these enzymes have demonstrated promising preclinical activity, their clinical translation may be limited by toxicity, insufficient blood–brain barrier penetration, and dose‐limiting adverse effects [13, 34, 35, 36, 37, 38]. We therefore propose that simultaneous modulation of DPP4, GK, BACE1, GSK3β, AChE, and TACE may disrupt the pathogenic feed‐forward loop between metabolic dysfunction and neurodegeneration, providing a rational basis for developing safer, multi‐target natural compounds with potential relevance to T2DM‐ and AD‐associated processes while potentially delaying the onset or progression of AD.

Medicinal plants have been widely used in ethnomedicine for their structurally diverse bioactive compounds, whose relatively promiscuous target profiles can modulate disease pathways via multi‐target mechanisms [39, 40, 41, 42]. In line with this concept, previous work demonstrated that an aqueous extract prepared from a combination of *Sclerocarya birrea*, *Nauclea latifolia*, and *Piper longum* (the SNP mixture) has been reported to display antidiabetic activity together with neuroprotective properties in a fructose/streptozotocin‐induced T2DM rat model with cognitive decline, improving glycemic control and memory performance [43]. Chemical characterization by HPLC–MS indicated seven major metabolites in this SNP mixture—7‐hydroxycadalene (7HC), linolenic acid (LIA), euparvic acid (EA), lepidimoic acid (LEA), mangiferin (MNG), quercetin pentamethyl ether (QR‐PME), and xanthomicrol (XNM)—as putative contributors to these dual antidiabetic and neuroprotective actions.

Structure‐based drug design (SBDD) has increasingly relied on molecular docking and molecular dynamics (MD) simulations to characterize how phytochemicals interact with protein targets involved in insulin resistance, amyloid aggregation, and neuroinflammation in complex disorders such as T2DM and AD [5, 44, 45, 46, 47]. Molecular docking provides initial molecular predictions of binding poses, relative affinities, and key residue contacts of small molecules against target receptors [48, 49, 50], while MD simulations refine these poses by explicitly accounting for protein and ligand flexibility and solvent effects, thereby offering a more realistic description of the structural and thermodynamic evolution of protein–ligand complexes over time [51, 52].

Docking‐based hit discovery, however, may over‐prioritize chemotypes that subsequently underperform once human metabolism and pharmacokinetics are considered, necessitating systematic evaluation of selected hits—covering absorption, distribution, metabolism, excretion, and toxicity (ADMET) [53, 54]. Because pharmacologically active small molecules—including conventional drugs and plant‐derived constituents—affect the phenotype through selective interactions with defined macromolecular partners, delineating their wider target landscape is essential for mechanistic interpretation and for reducing the likelihood of unintended off‐target effects. Accordingly, integrating ligand‐based target prediction tools such as SwissTargetPrediction [55, 56] and in silico ADMET analyses into SBDD workflows enables docking‐identified hits to be assessed not only for affinity toward a single intended target but also for their predicted polypharmacological target spectrum and pharmacokinetic behavior [55, 56, 57], thereby facilitating the selection of compounds with mechanistically relevant polypharmacology while filtering out candidates with poor developability or high off‐target risk at an early stage [55, 56, 57].

**FIGURE 1:**
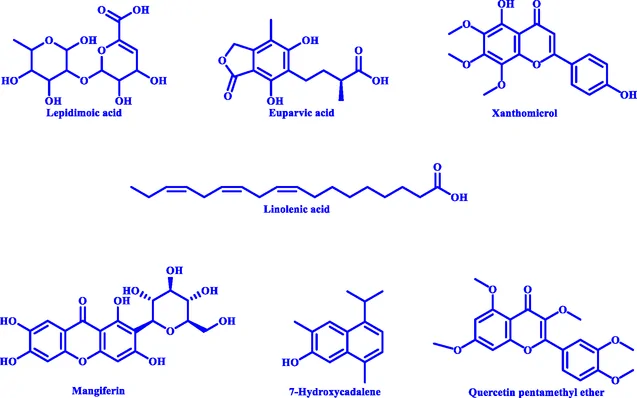
Chemical structures of the compounds. The 2D structures were generated using ChemOffice software (version 19.1).

Thus, we computationally screened the major phytochemicals identified in the SNP mixture (Figure 1) against six enzymes relevant to T2DM and AD to characterize their putative multi‐target interaction profiles. The workflow integrated molecular docking and MM‐GBSA rescoring with three independently initiated 100‐ns MD simulations of prioritized complexes. The structural and energetic behavior was evaluated using RMSD, RMSF, radius of gyration, potential energy profiles, and post‐MD MM‐GBSA calculations, complemented by per‐residue energy decomposition. Drug‐likeness and ADMET assessments were used to characterize the physicochemical and predicted pharmacokinetic properties of the phytochemicals, while ligand‐based target prediction explored the putative polypharmacology of the principal computational hit. By integrating predicted binding energetics, interaction stability, developability, and target profiles, this study aimed to prioritize experimentally testable phytochemical candidates and generate mechanistically grounded hypotheses concerning the interconnected pathologies of T2DM and AD.

## 2 Results and Discussion

The physicochemical identifiers of the screened phytochemicals are summarized in Table 1.

**TABLE 1:** PubChem compound identifier numbers, molecular weights, and molecular formulas of the phytochemicals that were docked against AChE, BACE1, DPP4, glucokinase, GSK3β, and TACE.

No	Name of compound	PubChem CID	Molecular weight, g/mol	Molecular formula
1	7‐Hydroxycadalene	608115	214.30	C_15_H_18_O
2	Linolenic acid	5280934	278.40	C_18_H_30_O_2_
3	Euparvic acid	45481542	280.27	C_14_H_16_O_6_
4	Mangiferin	5281647	422.30	C_19_H_18_O_11_
5	Lepidimoic acid	4479101	322.26	C_12_H_18_O_10_
6	Quercetin pentamethyl ether	97332	372.40	C_20_H_20_O_7_
7	Xanthomicrol	73207	344.30	C_18_H_16_O_7_

Table 2 shows the molecular docking scores, MM‐GBSA‐derived free energy estimates, and the interaction fingerprints of the top‐ranked docked poses of the major phytochemicals of the SNP mixture and the co‐crystallized inhibitors/activators against AChE, BACE1, DPP4, glucokinase (GK), GSK3β, and TACE in their active sites. In comparing phytochemicals with the positive (reference) controls for each enzyme, we assigned greater weight to the MM‐GBSA binding free energies (Δ*G*_MM‐GBSA_) than to the docking scores (Δ*G*_docking_) since the MM‐GBSA calculations incorporate implicit aqueous solvent effects on ligand–protein binding and are therefore generally considered to provide a more physically realistic estimate of binding energetics.

**TABLE 2:** Docking and MM‐GBSA binding free energy estimates and active‐site interaction profiles of SNP‐mixture phytochemicals and reference ligands across six human targets (AChE, BACE1, DPP4, GK, GSK3β, and TACE).

Compound	Molecular weight, g/mol	Receptor	Δ*G*_docking_, kcal/mol	Δ*G*_MM‐GBSA_, kcal/mol	Classical H‐bond	Nonclassical H‐bond (carbon‐hydrogen, pi‐donor)		Hydrophobic	Electrostatic (attractive charge, salt bridge, pi‐cation, pi‐anion)	Miscellaneous (fluorine, pi‐sulfur)
								*π*‐sigma, alkyl, *π*‐alkyl and *π*‐*π* stacked interactions		
Compound 5b (reference inhibitor)	532.10	AChE	−12.86	−68.29	—	—		Tyr70 (5.19 Å), Trp84 (3.57 Å, 4.12 Å, 4.26 Å, 5.35 Å), Trp94 (4.74 Å), Tyr121 (4.67 Å), Tyr242 (5.04 Å), Trp279 (4.01 Å, 4.04 Å, 4.18 Å), Phe290 (5.23 Å), Phe330 (3.68 Å, 3.92 Å, 5.18 Å, 5.21 Å), Tyr334 (4.59 Å), Trp432 (3.47 Å, 3.84 Å), Met436 (5.33 Å), Ile439 (3.96 Å)	—	—
7HC	214.30	AChE	−10.73	−37.91	His440 (2.39 Å)	—		*Trp84* (3.64, 3.94, 4.16, 4.70, 5.44 Å), *Phe330* (3.78, 4.06, 5.04 Å), *Tyr334* (4.66 Å)	—	—
EA	280.27	AChE	−9.68	−46.16	Gly119 (2.74 Å), Ser200 (2.09 Å), *Tyr334* (2.36 Å), His440 (2.69 Å)	—		*Trp84* (3.62 Å, 4.70 Å), *Phe330* (3.79 Å)	His440 (2.89 Å)	—
LEA	322.26	AChE	−8.01	−42.06	*Tyr121* (2.09 Å), *Trp84* (2.07 Å), Gly117 (2.60 Å), Asn85 (2.40 Å)	His440 (2.95 Å), *Tyr334* (3.85 Å)		—	—	—
LIA	278.40	AChE	−8.12	−46.88	Phe288 (2.55 Å)	—		*Trp84* (3.76 Å, 4.36 Å, 4.46 Å, 5.25 Å, 5.26 Å), *Phe330* (3.87 Å, 4.21 Å, 4.77 Å, 5.07 Å), *Tyr334* (4.46 Å, 4.65 Å), *Trp432* (3.79 Å, 3.86 Å), *Met436* (5.35 Å), *Ile439* (4.33 Å), Tyr442 (5.34 Å)	—	—
MNG	422.30	AChE	−6.81	−38.81	Tyr70 (2.75 Å), Asp72 (2.25 Å, 2.63 Å), Asn85 (2.78 Å), Ser286 (2.15 Å), *Tyr334* (2.00 Å)	—		—	—	—
QR‐PME	372.40	AChE	−7.58	−42.96	—	Asp72 (3.46 Å), Ser81 (3.77 Å), *Trp84* (3.72 Å), Asn85 (3.58 Å), *Tyr121* (2.47 Å)		Trp84 (4.86 Å), *Tyr334* (4.64, 5.06 Å)	Asp72 (4.46 Å)	—
XNM	344.30	AChE	−9.21	−45.87	Asp72 (3.03 Å), Ser122 (2.10 Å)	Gln69 (3.39 Å), *Trp84* (3.18 Å), Gly118 (3.55 Å)		Trp84 (3.64, 4.61, 4.88, 5.06 Å), *Phe330* (3.80, 4.48 Å), *Tyr334* (3.97 Å), *Ile439* (5.41 Å)	—	—
Inhibitor (Aminothiazine)	390.40	BACE1	−8.04	−32.37	—	Ser35 (3.37 Å)		Tyr71 (4.11 Å, 4.49 Å), Ile110 (3.80 Å)	Asp32 (4.88 Å)	Gly11 (3.01 Å)
7HC	214.30	BACE1	−7.50	−26.95	—	—		*Tyr71* (3.69 Å, 4.14 Å)	—	—
EA	280.27	BACE1	−6.77	−29.74	*Tyr71* (2.02 Å)	—		Tyr71 (3.87 Å, 4.19 Å)	*Asp32* (4.61 Å)	—
LEA	322.26	BACE1	−6.77	−29.13	*Asp32* (2.09 Å), Lys107 (2.69 Å), Gly230 (2.00 Å), Thr232 (2.31 Å)	—		*Tyr71* (3.97 Å)	—	—
LIA	278.40	BACE1	−6.36	−38.18	Thr232 (2.79 Å)	—		Leu30 (4.90 Å), *Tyr71* (4.03 Å, 4.51 Å, 5.01 Å), Phe108 (4.85 Å), Trp115 (4.85 Å), Ile118 (4.48 Å)	—	—
MNG	422.30	BACE1	−8.47	−40.69	Gly34 (2.56 Å), Arg235 (2.79 Å), Thr329 (2.80 Å)	Arg235 (3.42 Å)		*Tyr71* (5.31, 5.32 Å)	—	—
QR‐PME	372.40	BACE1	−7.49	−37.46	Thr232 (2.58, 2.60 Å)	Lys107 (3.77 Å), Phe108 (3.38 Å)		Val69 (5.41 Å), *Tyr71* (3.88, 4.37, 4.64 Å), Trp76 (4.47 Å), Ile118 (5.25 Å)	—	—
XNM	344.30	BACE1	−7.78	−35.68	Thr232 (2.53 Å)	Thr232 (3.72 Å)		*Tyr71* (3.69 Å)	*Asp32* (4.75 Å)	—
Inhibitor (Vildagliptin)	303.40	DPP4	−6.95	−38.71	Arg125 (2.87 Å), Asn710 (2.63 Å), His740 (2.81 Å)	Tyr662 (2.96 Å)		Phe357 (4.75 Å, 4.95 Å), Tyr662 (5.22 Å), *Tyr666* (4.40 Å)	—	—
7HC	214.30	DPP4	−6.56	−18.43	—	—		*Phe357* (3.78 Å, 4.45 Å, 4.88 Å), *Tyr666* (4.47 Å)	—	—
EA	280.27	DPP4	−7.02	−30.00	Glu205 (2.66 Å), *Arg125* (2.67 Å), *Tyr662* (2.64 Å), *Asn710* (2.52 Å), *His740* (2.73 Å)	—		*Phe357* (4.91 Å)	*Arg125* (5.32 Å), His740 (4.19 Å)	—
LEA	322.26	DPP4	−6.90	−32.18	Glu206 (2.63, 2.64 Å)	Glu205 (3.49 Å)		—	*Arg125* (2.32, 2.35 Å), *His740* (4.55 Å)	—
LIA	278.40	DPP4	−5.98	−32.29	Arg669 (2.07 Å, 2.42 Å)	—		*Phe357* (4.69 Å, 4.72 Å, 5.47 Å), Val656 (4.44 Å), Trp659 (4.72 Å), *Tyr662* (3.64 Å, 5.38 Å), *Tyr666* (4.18 Å, 4.32 Å, 5.25 Å)	Arg669 (4.79 Å)	—
MNG	422.30	DPP4	−7.28	−33.54	*Arg125* (2.55 Å), His126 (2.77 Å), *Tyr666* (2.96 Å), *Asn710* (2.29 Å)	—		*Tyr666* (4.54 Å)	—	—
QR‐PME	372.40	DPP4	−6.75	−35.76	Ser630 (2.06 Å)	Glu205 (3.19 Å, 3.56 Å)		*Phe357* (4.48 Å, 5.02 Å), Tyr547 (4.61 Å), Val656 (5.15 Å), *Tyr662* (3.95 Å), Val711 (5.10 Å), *His740* (4.31 Å)	Glu205 (4.45 Å)	—
XNM	344.30	DPP4	−6.52	−27.96	*Arg125* (2.38 Å, 2.95 Å)	—		*Phe357* (4.15 Å, 5.18 Å), Tyr547 (5.33 Å)	—	—
Activator (Compound 72)	519.64	Glucokinase	−9.21	−59.46	Arg63 (2.68 Å, 3.21 Å)	—		Val62 (4.56 Å), Arg63 (4.89 Å), Pro66 (4.74 Å), Ile149 (5.06 Å), Met200 (4.79 Å), Ile201 (4.77 Å, 5.12 Å), Tyr204 (3.97 Å, 4.16 Å, 5.05 Å), Met225 (4.66 Å), Val442 (4.72 Å), Val445 (3.92 Å)	—	Tyr204 (5.21 Å), Tyr205 (5.84 Å)
7HC	214.30	Glucokinase	−7.08	−31.78	—	—		*Pro66* (5.21 Å), *Met200* (4.78 Å), *Ile201* (5.24 Å), *Tyr204* (3.77 Å, 4.19 Å, 4.83 Å), *Met225* (4.56 Å)	—	—
EA	280.27	Glucokinase	−6.89	−27.60	—	*Val62* (3.54 Å), Ser64 (3.38 Å)		*Ile201* (5.45 Å), *Val445* (5.29 Å)	—	—
LEA	322.26	Glucokinase	−6.24	−39.60	—	—		*Tyr204* (3.80 Å)	—	—
LIA	278.40	Glucokinase	−6.45	−42.87	—	—		*Val62* (4.71 Å), *Pro66* (5.19 Å, 5.23 Å), *Ile149* (4.19 Å), *Met200* (5.35 Å), *Ile201* (4.50 Å, 5.28 Å), Tyr203 (4.75 Å, 4.03 Å), *Tyr204* (4.02 Å, 4.75 Å, 5.45 Å), *Met225* (4.55 Å), Leu441 (5.32 Å), *Val442* (4.43 Å, 4.44 Å), *Val445* (4.33 Å, 4.83 Å), Ala446 (3.90 Å)	Arg240 (4.77 Å)	—
MNG	422.30	Glucokinase	−8.78	−31.99	*Arg63* (2.54 Å, 1.86 Å)	—		*Tyr204* (4.33 Å, 4.36 Å, 5.65 Å)	—	—
QR‐PME	372.40	Glucokinase	−6.85	−39.93	—	—		*Ile201* (3.46 Å), *Tyr204* (4.04 Å, 4.87 Å, 5.12 Å), *Val442* (3.98 Å), *Val445* (3.77 Å)	—	—
XNM	344.30	Glucokinase	−7.17	−35.83	Tyr204 (2.30 Å)	*Pr*o66 (3.62 Å)		*Ile201* (5.37 Å), *Tyr204* (4.12 Å), *Met225* (5.02 Å)	—	—
Inhibitor (BRD0705)	321.41	GSK3β	−7.59	−31.37	Val131 (2.76 Å)	—	Ile62 (3.66 Å), Val67 (4.52 Å, 4.70 Å), Ala80 (3.66 Å, 4.52 Å, 4.72 Å), Tyr130 (5.12 Å), Leu184 (3.78 Å)		—	—
7HC	214.30	GSK3β	−6.74	−23.15	—	—	*Val67* (3.74 Å, 4.58 Å, 5.42 Å), *Ala80* (4.50 Å, 4.97 Å, 5.37 Å), Val107 (3.91 Å), Leu128 (4.02 Å), *Leu184* (5.08 Å), Cys195 (4.27 Å, 4.76 Å)		—	—
EA	280.27	GSK3β	−6.49	−32.65	—	—	*Ala80* (4.20 Å), Cys195 (4.88 Å)		—	—
LEA	322.26	GSK3β	−5.94	−29.10	Asp129 (2.34 Å), Pro132 (2.53 Å)	—	—		—	—
LIA	278.40	GSK3β	−5.48	−33.83	—	—	*Val67* (3.80 Å), *Ala80* (4.16 Å, 4.31 Å, 4.73 Å), Val107 (4.55 Å), Leu128 (4.54 Å, 4.84 Å), *Tyr130* (4.51 Å), *Leu184* (4.65 Å), Cys195 (4.24 Å, 4.38 Å, 4.99 Å)		—	—
MNG	422.30	GSK3β	−7.25	−31.98	Asp196 (2.99 Å)	Arg137 (3.59 Å)	*Val67* (5.48 Å), *Ala80* (4.26 Å, 5.15 Å), *Tyr130* (5.42 Å)		—	Cys195 (4.93 Å, 5.84 Å)
QR‐PME	372.40	GSK3β	−6.62	−36.44	—	Arg137 (3.71 Å), Gln181 (3.75 Å)	*Ile62* (4.82 Å, 5.34 Å), *Ala80* (3.99 Å), Val107 (3.72 Å), Leu128 (4.00 Å), *Tyr130* (5.64 Å), *Leu184* (5.06 Å, 5.07 Å), Cys195 (3.98 Å)		—	—
XNM	344.30	GSK3β	−7.12	−31.71	Arg137 (2.27 Å)	Thr134 (3.42 Å)	*Ala80* (4.38 Å), *Leu184* (3.77 Å), Cys195 (5.22 Å)		—	—
Inhibitor (Hydantoin ligand)	290.27	TACE	−7.51	−38.32	Gly349 (2.57 Å), Pro433 (3.20 Å), Leu348 (2.47 Å)	Glu394 (3.62 Å), His411 (3.71 Å), Val436 (3.15 Å)	Leu397 (4.60 Å), Val398 (4.44 Å, 5.21 Å), His401 (3.91 Å), Ala435 (3.42 Å, 3.69 Å)		—	—
7HC	214.30	TACE	−6.32	−29.22	—	—	Thr347 (3.99 Å), *His401* (3.51 Å), *Ala435* (3.96 Å, 4.90 Å)		—	—
EA	280.27	TACE	−7.47	−35.67	—	—	—		*His401* (3.44 Å, 3.98 Å)	—
LEA	322.26	TACE	−6.72	−34.33	Gly346 (2.91 Å), *Leu348* (2.20 Å), *Pro433* (2.09 Å)	—	—		*His401* (2.54 Å), His405 (3.02 Å), *His411* (4.10 Å),	—
LIA	278.40	TACE	−5.55	−36.45	—	—	*Leu348* (5.22 Å, 5.29 Å), *Leu397* (4.44 Å), *Val398* (5.00 Å, 5.20 Å), *His401* (4.19 Å), *Ala435* (3.75 Å, 3.95 Å, 4.11 Å), *Val436* (4.63 Å)		His401 (2.02 Å), *His411* (2.05 Å), His405 (3.71 Å)	—
MNG	422.30	TACE	−7.61	−33.76	Met345 (2.49 Å), *His401* (1.88 Å), His405 (2.05 Å), *His411* (2.21 Å)	His411 (3.44 Å)	Gly346 (3.97 Å), Thr347 (3.97 Å), *Ala435* (5.01 Å)		—	—
QR‐PME	372.40	TACE	−7.58	−48.15	Leu348 (2.78 Å), *His401* (2.66 Å), His405 (2.92 Å)	Thr347 (3.76 Å), Ala351 (3.79 Å), Tyr432 (2.49 Å), *Val436* (3.33 Å)	Val314 (5.18 Å), *Leu348* (5.16 Å, 5.24 Å), Leu350 (4.48 Å, 4.78 Å), *Leu397* (4.89 Å), *Val398* (4.62 Å, 4.32 Å), His401 (3.92 Å, 4.20 Å), His405 (3.90 Å), *Ala435* (3.47 Å, 3.60 Å, 4.14 Å)		Glu402 (4.75 Å)	—
XNM	344.30	TACE	−8.00	−37.53	*His401* (2.36 Å), His405 (2.52 Å), *His411* (2.45 Å)	Thr347 (3.53 Å), *Gly349* (3.77 Å), Glu402 (3.42 Å, 3.48 Å)	*Val398* (5.15 Å), His401 (3.87 Å), *Ala435* (3.51 Å)		His401 (4.20 Å)	—

### 2.1 Molecular Docking and MM‐GBSA Analysis of Phytochemical Interactions Against AChE

**FIGURE 2:**
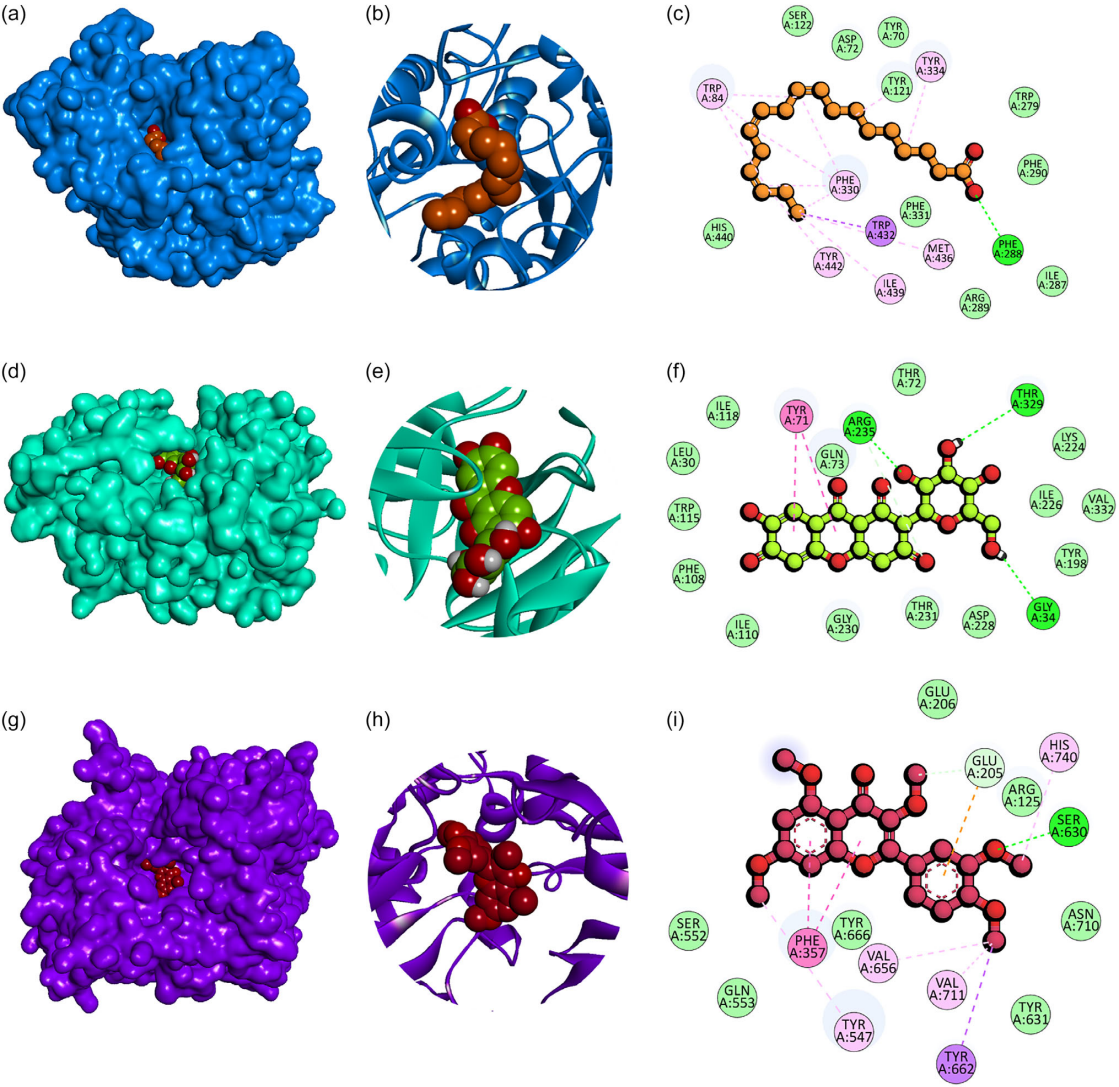
Top‐ranked docking poses of linolenic acid (LIA), mangiferin (MNG), and quercetin pentamethyl ether (QR‐PME) in the catalytic sites of AChE, BACE1, and DPP4, respectively. From left to right: (a,d,g) whole‐protein surface representation of the protein–ligand interaction; (b,e,h) magnified three‐dimensional overviews of the ligand conformations in the active sites (enzyme shown as a ribbon; phytochemical in CPK representation); and (c,f,i) two‐dimensional amino acid interaction networks. Panels (a–c) depict the AChE–LIA complex; (d–f) the BACE1–MNG complex; and (g–i) the DPP4–QR‐PME complex. Green dashed lines in the rightmost panels (c,f,i) indicate conventional hydrogen (H) bonds; light pastel‐green dashed lines indicate nonconventional C─H bonds; light and dark purple dashed lines denote hydrophobic contacts; and orange dashed lines indicate electrostatic interactions. Images were generated using Discovery Studio v16. CPK, Corey–Pauling–Koltun.

The reference co‐crystal inhibitor showed the lowest (most negative) docking score (Δ*G*_docking_ = −12.86 kcal/mol) and a binding free energy (Δ*G*_MM‐GBSA_ = −68.29 kcal/mol) with numerous hydrophobic contacts with Tyr70, Trp84, Trp94, Tyr121, Tyr242, Trp279, Phe290, Phe330, Tyr334, Trp432, Met436, and Ile439 (Table 2). Among the phytochemicals, LIA yielded a favorable docking score (Δ*G*_docking_ = –8.12 kcal/mol) with the most favorable MM‐GBSA–estimated binding free energy (Δ*G*_MM‐GBSA_ = –46.88 kcal/mol) against AChE, forming a single hydrogen (H) bond with Phe288 and extensive hydrophobic contacts with Trp84, Phe330, Tyr334, Trp432, Met436, Ile439, and Tyr442 and engaging in numerous van der Waals contacts with Tyr70, Asp72, Tyr121, Ser122, Trp279, Ile287, Arg289, Phe290, Phe331, and His440. Notably, the residues most frequently contacted by LIA (Trp84, Phe330, Tyr334, Trp432, Met436, and Ile439) substantially overlap with those contacted by the co‐crystallized inhibitor, a pattern consistent with occupancy of the same binding pocket and potentially competitive binding behavior. EA ranked second (Δ*G*_docking_ = −9.68 kcal/mol; Δ*G*_MM‐GBSA_ = −46.16 kcal/mol), forming H‐bonds with Gly119, Ser200, Tyr334, and His440, hydrophobic contacts with Trp84 and Phe330, and showing an additional electrostatic interaction with His440 (Figure 2a–c and Table 2).

### 2.2 Molecular Docking and MM‐GBSA Analysis of Phytochemical Interactions Against BACE1

Aminothiazine, the co‐crystallized BACE1 inhibitor, showed highly favorable interactions in the active site (Δ*G*_docking_ = −8.04 kcal/mol; Δ*G*_MM‐GBSA_ = −32.37 kcal/mol), characterized by a nonclassical H‐bond with Ser35, hydrophobic contacts with Tyr71 and Ile110, and an electrostatic interaction with Asp32. Relative to aminothiazine, MNG yielded both a more favorable docking score and MM‐GBSA estimate (Δ*G*_docking_ = −8.47 kcal/mol; Δ*G*_MM‐GBSA_ = −40.69 kcal/mol), forming multiple classical/nonclassical H‐bonds with Gly34, Arg235, and Thr329, together with a hydrophobic contact at Tyr71—an active‐site residue also contacted by the inhibitor (Figure 2d–f, Table 2)—indicative of a possible competitive binding mode. LIA also produced a more favorable MM‐GBSA binding free energy estimate but a less favorable docking score than aminothiazine (Δ*G*_docking_ = −6.36 kcal/mol; Δ*G*_MM‐GBSA_ = −38.18 kcal/mol), forming an H‐bond with Thr232 and hydrophobic contacts with Leu30, Tyr71, Phe108, Trp115, and Ile118. Notably, all seven ligands formed nonbonded contacts with Tyr71 of BACE1, implicating this residue as a recurrent interaction hotspot whose occupancy may contribute to the inhibitory potential (Table 2).

### 2.3 Molecular Docking and MM‐GBSA Analysis of Phytochemical Interactions Against DPP4

The co‐crystallized inhibitor vildagliptin showed favorable interactions within the DPP4 active site (Δ*G*_docking_ = −6.95 kcal/mol; Δ*G*_MM‐GBSA_ = −38.71 kcal/mol), forming classical H‐bonds with Arg125, Asn710, and His740, a nonclassical H‐bond with Tyr662, and hydrophobic contacts with Phe357, Tyr662, and Tyr666. QR‐PME displayed a comparable docking interaction, although with a slightly less favorable docking score and MM‐GBSA estimate than vildagliptin (Δ*G*_docking_ = −6.75 kcal/mol; Δ*G*_MM‐GBSA_ = −35.76 kcal/mol), forming classical and nonclassical H‐bonds with Ser630 and Glu205, extensive hydrophobic contacts with Phe357, Tyr547, Val656, Tyr662, and His740, and an electrostatic interaction with Glu205 (Figure 2g–i, Table 2). Notably, QR‐PME repeatedly contacted Phe357, Tyr662, and His740, supporting a putative binding mode compatible with the occupancy of the reference ligand at the active site. Ranked second, MNG showed a more favorable docking score but a less favorable MM‐GBSA estimate than vildagliptin (Δ*G*_docking_ = −7.28 kcal/mol; Δ*G*_MM‐GBSA_ = −33.54 kcal/mol), yielding classical H‐bonds with Arg125, His126, Tyr666, and Asn710, alongside a hydrophobic contact with Tyr666 (Table 2).

### 2.4 Molecular Docking and MM‐GBSA Analysis of Phytochemical Interactions Against Glucokinase

Compound 72, the reference activator, showed highly favorable interactions with glucokinase (GK) (Δ*G*_docking_ = −9.21 kcal/mol; Δ*G*_MM‐GBSA_ = −59.46 kcal/mol) and established extensive hydrophobic contacts with Val62, Arg63, Pro66, Ile149, Met200, Ile201, Tyr204, Met225, Val442, and Val445.

**FIGURE 3:**
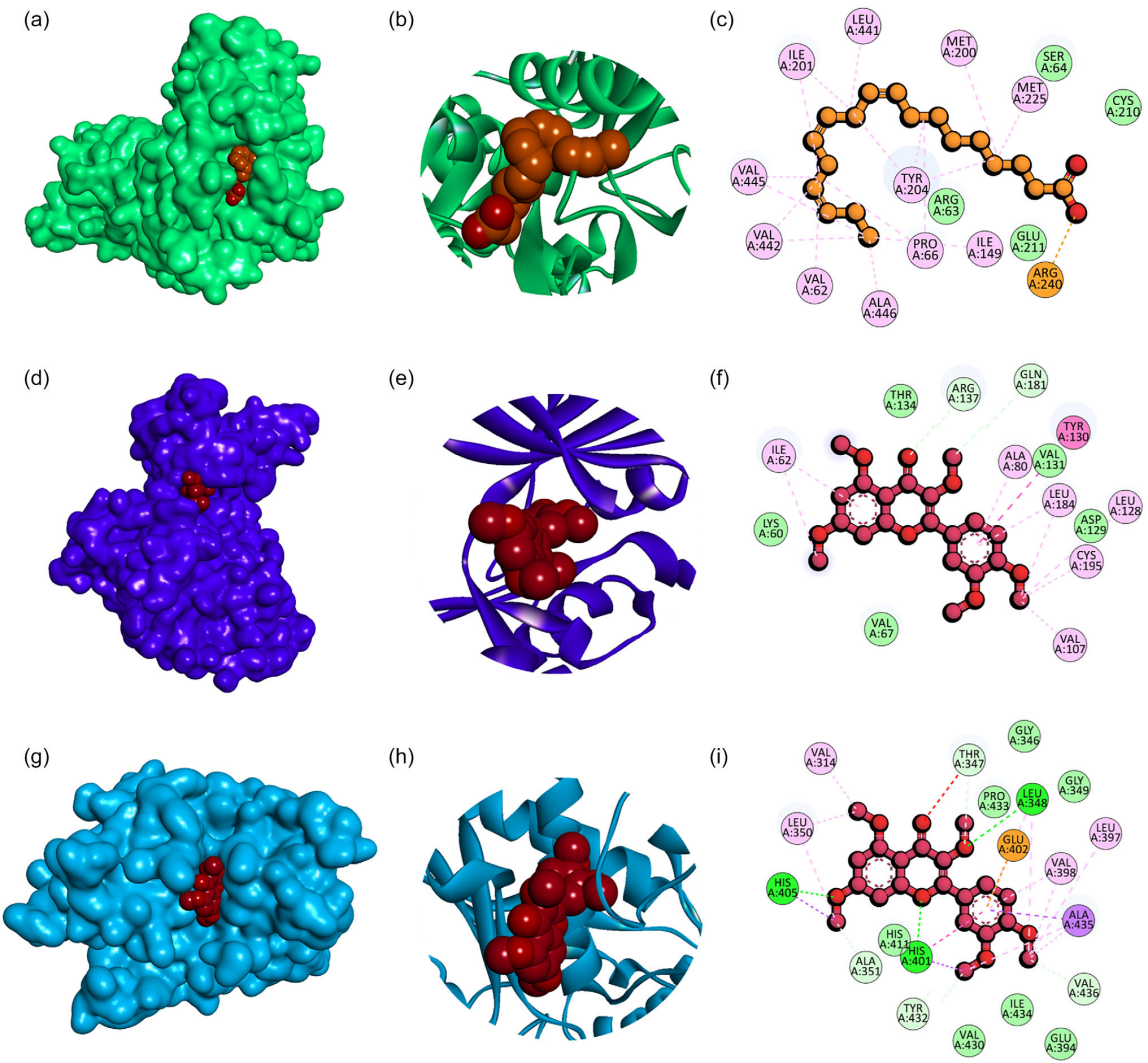
Top‐ranked docking poses of linolenic acid (LIA) and quercetin pentamethyl ether (QR‐PME) in the catalytic sites of Glucokinase, GSK3β, and TACE. From left to right: (a,d,g) whole‐protein surface representation of the protein–ligand interaction; (b,e,h) magnified three‐dimensional overviews of the ligand conformations in the active sites (enzyme shown as a ribbon; phytochemical in CPK representation); and (c,f,i) two‐dimensional amino acid interaction networks. Panels (a–c) depict the Glucokinase–LIA complex; (d–f) the GSK3β–QR–PME complex; and (g–i) the TACE–QR–PME complex. Green dashed lines in the rightmost panels (c,f,i) indicate conventional hydrogen (H) bonds; light pastel‐green dashed lines indicate nonconventional C─H bonds; light and dark purple dashed lines denote hydrophobic contacts; and orange dashed lines indicate electrostatic interactions. Images were generated using Discovery Studio v16. CPK, Corey–Pauling–Koltun.

Although its docking score was not the most favorable among the phytochemicals, LIA yielded the most negative MM‐GBSA estimate and was therefore selected as the hit compound against GK (Δ*G*_docking_ = −6.45 kcal/mol; Δ*G*_MM‐GBSA_ = −42.87 kcal/mol), forming hydrophobic contacts with Val62, Pro66, Ile149, Met200, Ile201, Tyr203, Tyr204, Met225, Leu441, Val442, Val445, and Ala446, together with an additional electrostatic interaction with Arg240. Notably, among the seven phytochemicals, LIA most consistently interacted with residues overlapping those contacted by the activator, closely mirroring the activator‐like interaction profile and supporting its candidacy as a potential GK activator (Figure 3a–c and Table 2).

### 2.5 Molecular Docking and MM‐GBSA Analysis of Phytochemical Interactions Against GSK3β

BRD0705, the co‐crystallized GSK3β inhibitor, showed a favorable interaction profile (Δ*G*_docking_ = −7.59 kcal/mol Δ*G*_MM‐GBSA_ = −31.37 kcal/mol), forming a classical H‐bond with Val131 and hydrophobic contacts with Ile62, Val67, Ala80, Tyr130, and Leu184. Across the phytochemical panel, docking scores spanned −5.48 to −7.25 kcal/mol; however, QR‐PME ranked first by MM‐GBSA, yielding a more favorable binding free energy (Δ*G*_MM‐GBSA_ = −36.44 kcal/mol) than BRD0705. QR‐PME formed nonclassical H‐bonds with Arg137 and Gln181 and extensive hydrophobic contacts with Ile62, Ala80, Val107, Leu128, Tyr130, Leu184, and Cys195 within the binding pocket. Notably, the QR‐PME residue interaction pattern—particularly involving Ile62, Ala80, Tyr130, and Leu184—closely resembles that of BRD0705, suggesting a putative competitive inhibition mode against GSK3β (Figure 3d–f and Table 2).

### 2.6 Molecular Docking and MM‐GBSA Analysis of Phytochemical Interactions Against TACE

Hydantoin, the co‐crystallized TACE inhibitor, showed favorable interactions in the catalytic pocket (Δ*G*_docking_ = −7.51 kcal/mol; Δ*G*_MM‐GBSA_ = −38.32 kcal/mol), forming classical and nonclassical H‐bonds with Leu348, Gly349, Glu394, His411, Pro433, and Val436 and hydrophobic contacts with Leu397, Val398, His401, and Ala435. Among the remaining ligands, QR‐PME yielded a similarly favorable docking score yet a markedly more favorable MM‐GBSA estimate than hydantoin (Δ*G*_docking_ = −7.58 kcal/mol Δ*G*_MM‐GBSA_ = −48.15 kcal/mol). QR‐PME formed classical and nonclassical H‐bonds with Thr347, Leu348, Ala351, His401, His405, Tyr432, and Val436, together with extensive hydrophobic contacts involving Val314, Leu348, Leu350, Leu397, Val398, His401, His405, and Ala435, and a single electrostatic interaction with Glu402. Notably, the interaction fingerprint of QR‐PME—centered on His401, Val436, Leu348, Leu397, Val398, and Ala435—closely matches that of the co‐crystallized inhibitor, supporting a possible competitive inhibition mode for QR‐PME against TACE (Figure 3g–i and Table 2).

### 2.7 Molecular Dynamics and MM‐GBSA Binding Free Energy Calculations of Major SNP‐Mixture Phytocompounds and Co‐Crystallized Ligands Against Target Proteins

**FIGURE 4:**
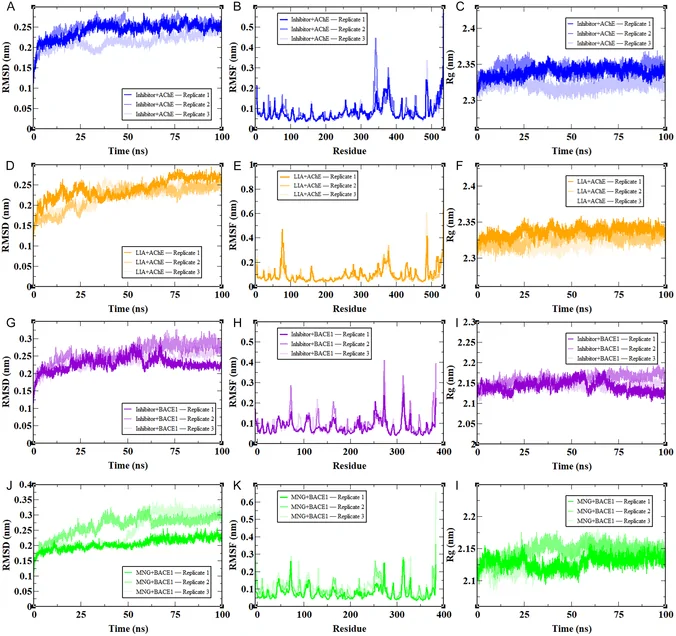
Comparative structural stability and flexibility of AChE and BACE1 complexes with their reference inhibitors and major phytochemicals across three independent 100‐ns molecular dynamics simulations. The left, middle, and right panels for each protein–ligand system present the time evolution of the root‐mean‐square deviation (RMSD) of protein Cα atoms, the residue‐wise root‐mean‐square fluctuation (RMSF), and the time‐dependent radius of gyration (*R*_g_), respectively. Panels (A–C) represent the AChE–compound 5b (reference inhibitor) complex (blue), panels (D–F) the AChE–linolenic acid (LIA) complex (orange), panels (G–I) the BACE1–aminothiazine (reference inhibitor) complex (purple), and panels (J–L) the BACE1–mangiferin (MNG) complex (green). Within each panel, the three curves correspond to three independently initiated 100‐ns simulation replicates (Replicates 1–3).

A focused visual inspection of each 100‐ns MD trajectory (*n* = 3) in PyMOL [58] indicated that compound 5b (co‐crystallized inhibitor) remained stable in the AChE catalytic pocket. Across three independent 100‐ns simulations, the AChE–compound 5b complexes showed mean RMSD values of 0.242, 0.249, and 0.214 nm and mean *R*_g_ values of 2.339, 2.343, and 2.320 nm, respectively, suggesting broadly comparable conformational behavior without a marked loss of protein compactness (Figure 4A–C). Mean Cα RMSF values were 0.081, 0.088, and 0.080 nm, with the largest fluctuations mainly localized to the terminal and loop regions of the enzyme. Per‐residue decomposition indicated favorable contributions from Trp84, Trp279, Phe290, Phe330, and Tyr334, whereas Glu199 contributed unfavorably (Figure S1). Across the three replicas, compound 5b formed 0.13 ± 0.002 H‐bonds and yielded a mean MM‐GBSA binding free energy of −59.59 ± 0.06 kcal/mol, calculated over the final 10 ns of each trajectory (Tables 3 and S1).

**TABLE 3:** Mean (± standard error) numbers of hydrogen bonds (H‐bonds) and MM‐GBSA binding free energies (Δ*G*; kcal/mol) obtained from three independent 100‐ns MD simulations of DPP4, GK, AChE, BACE1, GSK3β, and TACE in complex with their co‐crystallized inhibitors/activators or major phytochemicals from the SNP mixture.

Ligand	Receptor	Number of H‐bonds	Binding free energy, kcal/mol
Vildagliptin (reference inhibitor)	DPP4	1.72 ± 0.005	−28.01 ± 0.10
QR‐PME	DPP4	0.35 ± 0.003	−16.03 ± 0.06
Compound 72 (reference activator)	GK	1.23 ± 0.004	−56.71 ± 0.07
LIA	GK	0.38 ± 0.003	−41.97 ± 0.06
Compound 5b (reference inhibitor)	AChE	0.13 ± 0.002	−59.59 ± 0.06
LIA	AChE	1.05 ± 0.003	−31.73 ± 0.18
Aminothiazine (reference inhibitor)	BACE1	0.77 ± 0.004	−37.94 ± 0.06
MNG	BACE1	1.14 ± 0.006	−18.85 ± 0.06
BRD0705 (reference inhibitor)	GSK3β	0.48 ± 0.004	−30.05 ± 0.07
QR‐PME	GSK3β	0.41 ± 0.003	−30.07 ± 0.06
Hydantoin (reference inhibitor)	TACE	1.61 ± 0.006	−34.08 ± 0.06
QR‐PME	TACE	0.62 ± 0.003	−34.24 ± 0.07

*Note:* Values are reported as mean ± propagated SEM from three independent MD replicas. The SEM for each replica was calculated as SD/√n and combined as SEM_overall_ = √(SEM_1_^2^ + SEM_2_^2^ + SEM_3_^2^)/3. Configurations extracted at regular intervals from the final 10 ns of each trajectory were used for MM‐GBSA calculations.

The LIA–AChE complexes, across three independent 100‐ns simulations, showed mean RMSD values of 0.239, 0.220, and 0.222 nm and mean *R*_g_ values of 2.344, 2.329, and 2.321 nm, respectively, suggesting comparable conformational behavior without a marked loss of protein compactness (Figure 4D–F). Mean Cα RMSF values were 0.096, 0.085, and 0.091 nm, with the largest fluctuations mainly involving terminal and loop regions. Per‐residue binding free energy decomposition revealed predominantly favorable contributions from Trp84, Ile287, Phe330, Phe331, and Tyr334, whereas Glu199 and His440 contributed unfavorably (Figure S1). Across three independent replicas, LIA formed 1.05 ± 0.003 H‐bonds with AChE and yielded a mean MM‐GBSA binding free energy of −31.73 ± 0.18 kcal/mol, calculated over the final 10 ns of each trajectory (Tables 3 and S1).

Across three independent 100‐ns simulations, the BACE1–aminothiazine complexes showed mean RMSD values of 0.226, 0.257, and 0.235 nm and mean *R*_g_ values of 2.141, 2.159, and 2.141 nm, respectively, suggesting comparable conformational behavior without marked loss of protein compactness (Figure 4G–I). Mean Cα RMSF values were 0.082, 0.094, and 0.086 nm, with the largest fluctuations mainly localized to the loop regions. Per‐residue decomposition consistently indicated a favorable contribution from Tyr71, accompanied by smaller, replica‐dependent contributions from Leu30, Pro70, Trp76, Phe108, and Ile118 (Figure S2). Across the replicas, aminothiazine formed 0.77 ± 0.004 H‐bonds with BACE1 and yielded a mean MM‐GBSA binding free energy of −37.94 ± 0.06 kcal/mol, calculated over the final 10 ns of each trajectory (Tables 3 and S1).

Across three independent 100‐ns simulations, the BACE1–MNG complexes showed mean RMSD values of 0.205, 0.264, and 0.249 nm and mean *R*_g_ values of 2.128, 2.146, and 2.133 nm, respectively, suggesting moderate replica‐dependent conformational variability without a marked loss of protein compactness (Figure 4J–L). Mean Cα RMSF values were 0.075, 0.095, and 0.109 nm, with the largest fluctuations mainly localized to the loop and terminal regions. Per‐residue decomposition indicated favorable contributions from Tyr71 and Thr72, accompanied by replica‐dependent contributions from Pro70, Gln73, Ile126, Arg128, and Thr231 (Figure S2). Across the three replicates, MNG formed 1.14 ± 0.006 H‐bonds with BACE1 and yielded a mean MM‐GBSA binding free energy of −18.85 ± 0.06 kcal/mol over the final 10 ns (Tables 3 and S1).

**FIGURE 5:**
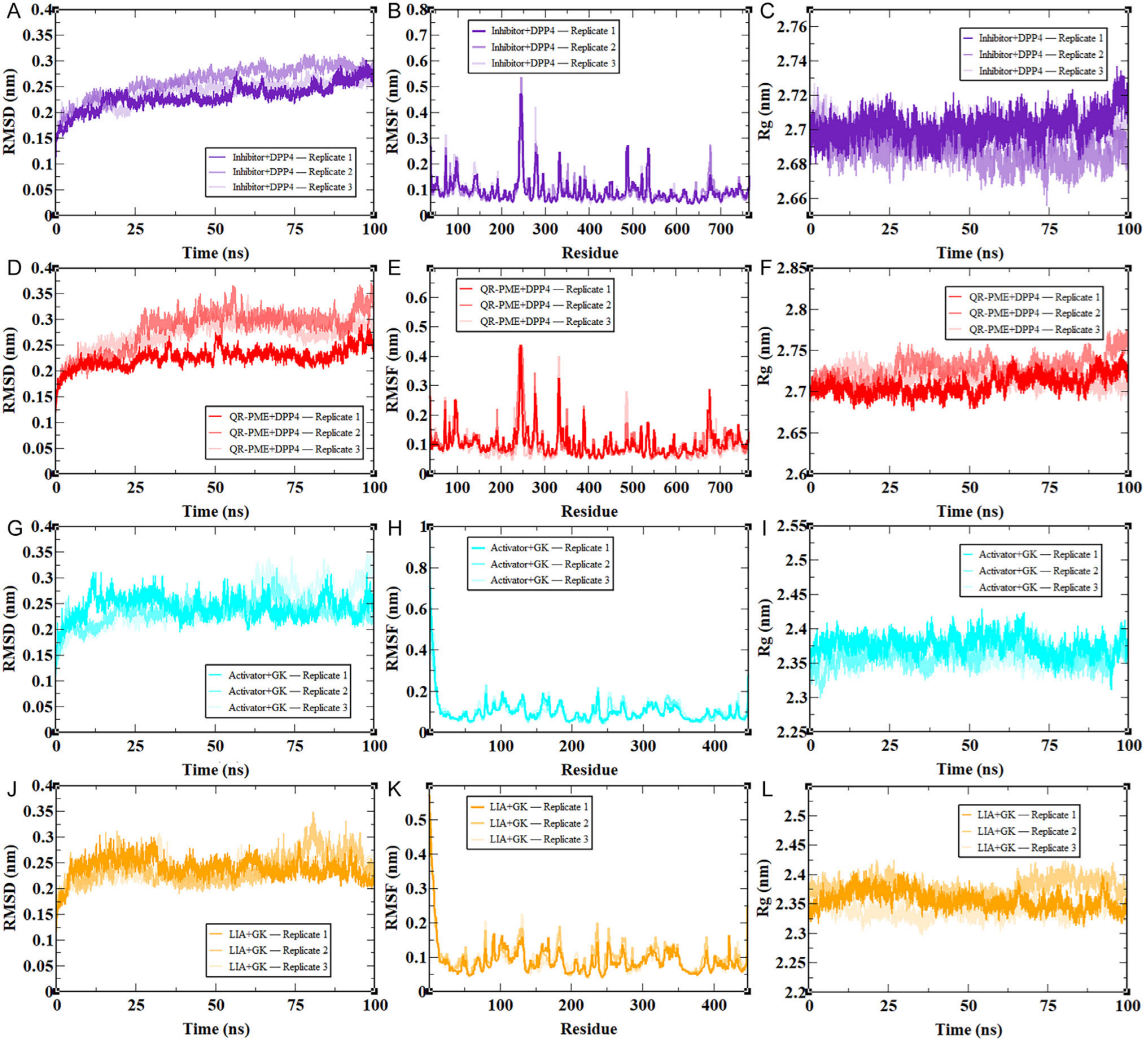
Comparative structural stability and flexibility of DPP4 and glucokinase (GK) complexes with their reference ligands and major phytochemicals across three independent 100‐ns molecular dynamics simulations. The left, middle, and right panels for each protein–ligand system show the time‐dependent root‐mean‐square deviation (RMSD) of protein Cα atoms, the residue‐wise root‐mean‐square fluctuation (RMSF), and the time‐dependent radius of gyration (*R*_g_), respectively. Panels (A–C) represent the DPP4–vildagliptin (reference inhibitor) complex (purple), panels (D–F) the DPP4–quercetin pentamethyl ether (QR‐PME) complex (red), panels (G–I) the GK–compound 72 (reference activator) complex (cyan), and panels (J–L) the GK–linolenic acid (LIA) complex (orange). Within each panel, the three curves correspond to three independently initiated 100‐ns simulation replicates (Replicates 1–3).

The DPP4–vildagliptin complexes, among three independent 100‐ns simulations, showed mean RMSD values of 0.230, 0.261, and 0.238 nm and mean *R*_g_ values of 2.702, 2.687, and 2.698 nm, respectively, suggesting broadly comparable conformational behavior without a marked loss of protein compactness (Figure 5A–C). Mean Cα RMSF values were 0.094, 0.092, and 0.090 nm, with the largest fluctuations mainly localized to the loop regions. Per‐residue decomposition consistently indicated favorable contributions from Glu205 and Tyr666, accompanied by replica‐dependent contributions from Glu206, Phe357, Tyr547, Trp629, and Tyr631 (Figure S3). Across the replicas, vildagliptin formed 1.72 ± 0.005 H‐bonds with DPP4 and yielded a mean MM‐GBSA binding free energy of −28.01 ± 0.10 kcal/mol over the final 10 ns (Tables 3 and S1).

Across three independent 100‐ns simulations, the DPP4–QR‐PME complexes showed RMSD values of 0.225, 0.280, and 0.270 nm and *R*_g_ values of 2.708, 2.728, and 2.716 nm, respectively, suggesting moderate replica‐dependent conformational variability without a marked loss of protein compactness (Figure 5D–F). Mean Cα RMSF values were 0.102, 0.105, and 0.091 nm, with the largest fluctuations mainly localized to loop regions in the protein. Per‐residue decomposition revealed replica‐dependent favorable contributions, notably from Arg125 and Trp629 in two replicas and from His740, Tyr547, Ser630, and His126 in individual trajectories; Lys122 contributed unfavorably in Replica 3 (Figure S3). Across the replicas, QR‐PME formed 0.35 ± 0.003 H‐bonds with DPP4 and yielded a mean MM‐GBSA binding free energy of −16.03 ± 0.06 kcal/mol over the final 10 ns, less favorable than vildagliptin (Tables 3 and S1).

Across three independent 100‐ns simulations, the GK–compound 72 complexes showed mean RMSD values of 0.242, 0.234, and 0.244 nm and mean *R*_g_ values of 2.376, 2.360, and 2.362 nm, respectively, suggesting broadly comparable conformational behavior without a marked loss of compactness of the protein structure (Figure 5G–I). Mean Cα RMSF values were 0.101, 0.099, and 0.105 nm, with the largest fluctuations predominantly confined to the terminal region of the protein. Per‐residue decomposition consistently indicated favorable contributions from Val62, Pro66, Ile201, Tyr204, and Val445, with no prominent unfavorable contribution among the displayed residues (Figure S4). Across the replicas, compound 72 formed 1.23 ± 0.004 H‐bonds with GK and yielded a mean MM‐GBSA binding free energy of −56.71 ± 0.07 kcal/mol over the final 10 ns (Tables 3 and S1).

Analyses of the three independent 100‐ns simulations of the GK–LIA complexes showed mean RMSD values of 0.239, 0.237, and 0.232 nm and mean *R*_g_ values of 2.356, 2.377, and 2.338 nm, respectively, suggesting broadly comparable conformational behavior without a marked loss of protein compactness (Figure 5J–L). Mean Cα RMSF values were 0.091, 0.099, and 0.094 nm, with the largest fluctuations predominantly confined to the protein terminal region. Per‐residue decomposition consistently indicated favorable contributions from Val62, Tyr204, and Val445, accompanied by smaller contributions from Pro66, Ile201, Met225, and Val442 (Figure S4). Across the replicas, LIA formed 0.38 ± 0.003 H‐bonds with GK and yielded a mean MM‐GBSA binding free energy of −41.97 ± 0.06 kcal/mol over the final 10 ns (Tables 3 and S1).

**FIGURE 6:**
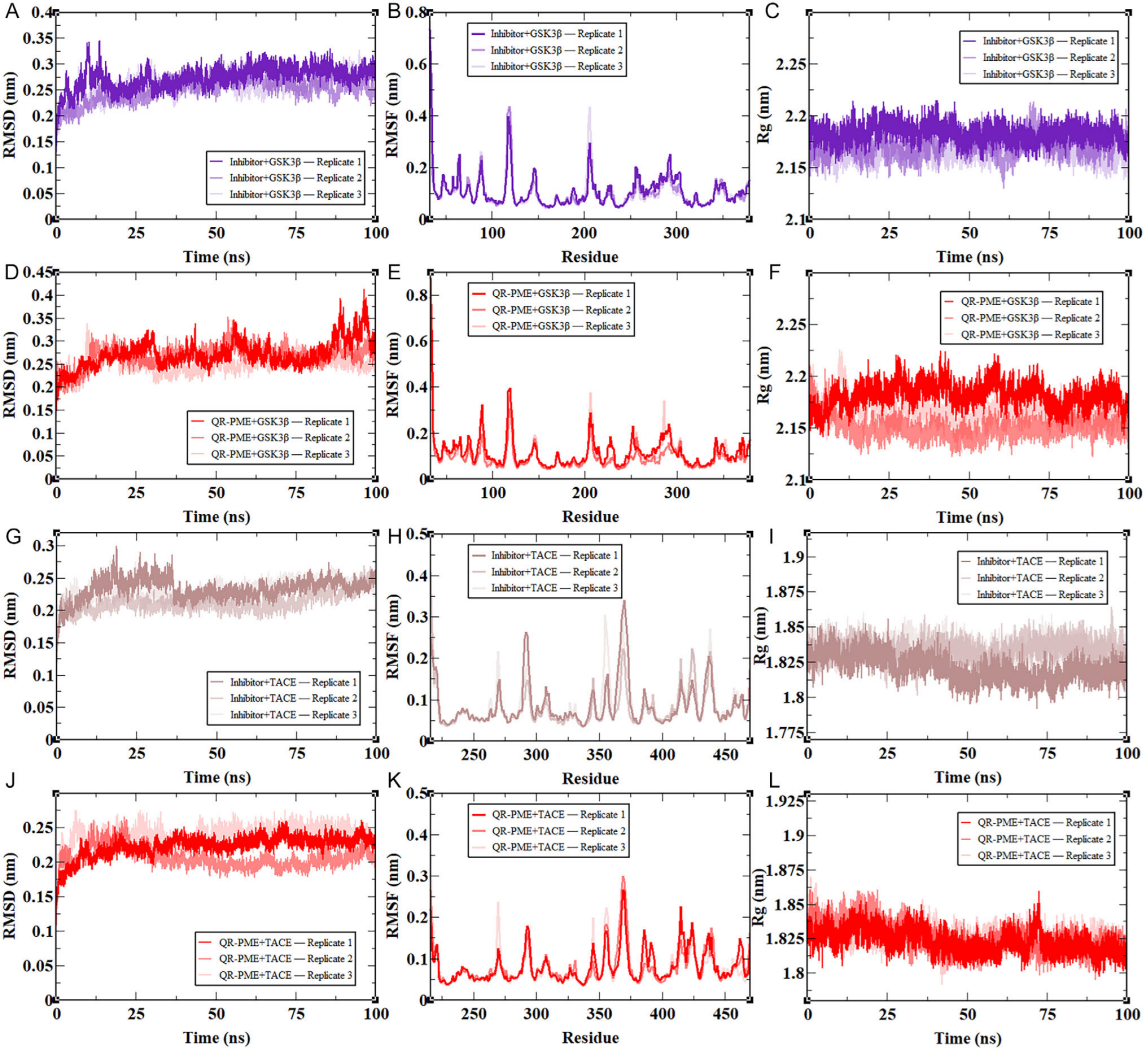
Comparative structural stability and flexibility of GSK3β and TACE complexes with their reference inhibitors and quercetin pentamethyl ether (QR‐PME) across three independent 100‐ns molecular dynamics simulations. The left, middle, and right panels for each protein–ligand system show the time‐dependent root‐mean‐square deviation (RMSD) of protein Cα atoms, the residue‐wise root‐mean‐square fluctuation (RMSF), and the time‐dependent radius of gyration (*R*_g_), respectively. Panels (A–C) represent the GSK3β–BRD0705 (reference inhibitor) complex (purple), panels (D–F) the GSK3β–QR–PME complex (red), panels (G–I) the TACE–hydantoin (reference inhibitor) complex (brown), and panels (J–L) the TACE–QR–PME complex (red). Within each panel, the three curves correspond to three independently initiated 100‐ns simulation replicates (Replicates 1–3).

BRD0705 remained associated with the GSK3β catalytic pocket during the three independent 100 ns simulations. The complexes sampled mean RMSD values of 0.272, 0.250, and 0.255 nm, whereas their mean *R*_g_ values remained within a narrow range of 2.166–2.183 nm, indicating limited changes in protein overall compactness (Figure 6A–C). Mean Cα RMSF values were 0.108, 0.103, and 0.103 nm, with the larger fluctuations mainly involving the terminal and loop regions of GSK3β. Per‐residue decomposition revealed favorable contributions from Ile62, Val67, and Leu184 in all replicas, with smaller contributions from Ala80, Leu128, Tyr130, and Thr134 (Figure S5). On average, BRD0705 formed 0.48 ± 0.004 H‐bonds with GSK3β, and its mean MM‐GBSA binding free energy over the final 10 ns was −30.05 ± 0.07 kcal/mol (Tables 3 and S1).

Across three independent 100‐ns simulations, the GSK3β–QR‐PME complexes showed mean RMSD values of 0.271, 0.267, and 0.248 nm and *R*_g_ values of 2.181, 2.150, and 2.166 nm, respectively, suggesting maintained compactness (Figure 6D–F). Mean Cα RMSF values were 0.117, 0.093, and 0.106 nm, with larger fluctuations mainly involving the terminal and loop regions of the protein. Per‐residue decomposition showed favorable contributions from Val67 and Leu184 across the replicas, with additional contributions from Phe64, Thr134, and Arg137 (Figure S5). QR‐PME formed 0.41 ± 0.003 H‐bonds and yielded a mean MM‐GBSA binding free energy of −30.07 ± 0.06 kcal/mol over the final 10 ns. The average free energy of binding of QR‐PME with GSK3β closely matched BRD0705 (−30.05 ± 0.07 kcal/mol), showing a comparable MM‐GBSA binding free energy estimate and highlighting it as a candidate for further exploration against GSK3β (Tables 3 and S1).

Hydantoin (inhibitor) remained accommodated within the TACE catalytic pocket during the three independent 100‐ns simulations. The complexes sampled mean RMSD values of 0.233, 0.212, and 0.231 nm, while their *R*_g_ values remained within a narrow range of 1.821–1.835 nm, suggesting limited changes in the global compactness of the protein structure (Figure 6G–I). Mean Cα RMSF values were 0.086, 0.078, and 0.088 nm, with the larger fluctuations mainly confined to loop regions in the protein. Per‐residue decomposition indicated favorable contributions from Leu348, Val398, Ile434, and Ala435, accompanied by Pro433 in two replicas; Glu402 contributed unfavorably in Replica 3 (Figure S6). On average, hydantoin formed 1.61 ± 0.006 H‐bonds with TACE and yielded a mean MM‐GBSA binding free energy of −34.08 ± 0.06 kcal/mol over the final 10 ns (Tables 3 and S1).

Across three independent 100‐ns simulations, the TACE–QR‐PME complexes showed mean RMSD values of 0.223, 0.206, and 0.237 nm and *R*_g_ values of 1.823, 1.826, and 1.826 nm, respectively, suggesting maintained compactness of the protein structure (Figure 6J–L). Mean Cα RMSF values were 0.082, 0.081, and 0.077 nm, with larger fluctuations mainly confined to the loop regions of the protein. Per‐residue decomposition identified favorable contributions from Leu348, Ile434, and Ala435 across the replicas, together with Val398, Pro433, and Val436; Glu402 contributed unfavorably to Replica 2 (Figure S6). QR‐PME formed 0.62 ± 0.003 H‐bonds and yielded an MM‐GBSA binding free energy of −34.24 ± 0.07 kcal/mol over the final 10 ns. The MM‐GBSA binding free energy of QR‐PME was slightly more favorable than that of hydantoin (−34.24 ± 0.07 versus −34.08 ± 0.06 kcal/mol), highlighting QR‐PME as a potential candidate phytochemical for interaction with TACE, warranting further investigation (Tables 3 and S1).

Finally, the potential energy profiles of all 12 protein–ligand systems showed no pronounced systematic drift across the three independent 100‐ns replicas (Figures S7–S9). Together with the closely distributed replicate means and small trajectory‐level SEM values (10.43–16.60 kJ mol^−1^) relative to the total system energies (Table S4), our findings showed statistically consistent energetic behavior of these protein–ligand systems before structural and MM‐GBSA analyses.

### 2.8 Physicochemical and Drug‐Likeness Prediction

Based on the integrated molecular docking and three independent 100‐ns MD simulations per protein–ligand complex, followed by MM‐GBSA binding free energy estimates, quercetin pentamethyl ether (QR‐PME) was selected as the primary phytochemical hit for further profiling. The physicochemical and drug‐likeness properties of the selected phytochemicals, with particular emphasis on QR‐PME, and those of the co‐crystallized/reference ligands were assessed using Lipinski's Rule of Five (Ro5), Veber‐type oral bioavailability criteria, and the Abbott/SwissADME bioavailability‐score model. These criteria generally favor compounds with MW ≤500 g/mol, LogP ≤5, ≤5 hydrogen‐bond donors, ≤10 hydrogen‐bond acceptors, TPSA ≤140 Å^2^, and ≤10 rotatable bonds, while a bioavailability score of 0.55 is commonly associated with a favorable probability of oral exposure [53, 59, 60, 61].

As shown in Table 4, the QR‐PME complied with Lipinski's Ro5 without any violation. It displayed a moderate TPSA of 76.36 Å^2^, a balanced consensus LogP_o_/_v_ of 3.04, six rotatable bonds, and a bioavailability score of 0.55, together indicating a physicochemical profile compatible with oral absorption. QR‐PME was also predicted to be moderately water soluble. Although it exceeded the more restrictive lead‐like molecular weight threshold of 350 g/mol, this represents an optimization consideration rather than an Ro5 violation.

**TABLE 4:** In silico physicochemical and drug‐likeness predictions of the compounds and the co‐crystallized inhibitors/activator.

Drug‐likeness assessment	Compounds							Co‐crystallized inhibitors/activator					
	7HC	LIA	EA	LEA	MNG	QR‐PME	XNM	AChE inhibitor	BACE1 inhibitor	GSK3β inhibitor	TACE inhibitor	DPP4 inhibitor	Glucokinase activator
# Rotatable bonds (rotors)	1	13	4	3	2	6	4	11	4	2	3	4	8
# H‐bond acceptors	1	2	6	10	11	7	7	4	6	2	4	4	7
# H‐bond donors	1	1	3	6	8	0	2	3	2	2	3	2	1
TPSA (Å^2^)	20.23	37.30	104.06	166.14	201.28	76.36	98.36	83.48	114.90	57.78	105.22	76.36	159.56
Consensus Log *P*_o/w_	4.02	5.66	1.83	−2.35	−0.77	3.04	2.42	5.48	2.45	3.46	0.49	1.33	3.80
Water solubility Log S (ESOL)	Moderately soluble	—	Soluble	Highly soluble	Soluble	Moderately soluble	Moderately soluble	Poorly soluble	Soluble	Moderately soluble	Very soluble	Soluble	Moderately soluble
Drug‐likeness (Lipinski's rule of five), # violations	Yes, 0	Yes, 0	Yes, 0	Yes, 1; NH or OH > 5	No, 2; N or O > 10, NH or OH > 5	Yes, 0	Yes, 0	Yes, 1; MW > 500	Yes, 0	Yes, 0	Yes, 0	Yes, 0	Yes, 1; MW > 500
Bioavailability score	0.55	—	0.56	0.11	0.17	0.55	0.55	0.55	0.55	0.55	0.55	0.55	0.55
Lead likeness, # violations	No, 2; MW < 250, XLOGP3 > 3.5	—	Yes	Yes	No, 1; MW > 350	No, 1; MW > 350	Yes	No, 3; MW > 350, Rotors > 7, XLOGP3 > 3.5	No, 1; MW > 350	No, 1; XLOGP3 > 3.5	Yes	Yes	No, 3; MW > 350, Rotors > 7, XLOGP3 > 3.5

The co‐crystallized inhibitors and reference activator all displayed bioavailability scores of 0.55 and generally complied with Lipinski's Ro5. Only the bulky AChE inhibitor and glucokinase activator showed a single violation associated with an MW > 500 g/mol. Most reference ligands did not satisfy the more stringent lead‐likeness thresholds, whereas the TACE and DPP4 inhibitors fulfilled these criteria (Table 4). In addition, the AChE inhibitor exceeded the commonly applied rotatable bond limit (11 > 10), while the glucokinase activator exceeded the standard TPSA threshold (159.56 > 140 Å^2^), demonstrating that physicochemical guideline deviations were also present among the co‐crystallized/reference compounds.

Overall, QR‐PME combined complete Ro5 compliance with acceptable polarity, lipophilicity, molecular flexibility, predicted solubility, and a bioavailability score of 0.55, supporting its prioritization for subsequent ADMET profiling. Its single lead‐likeness violation (MW > 350 g/mol) should be regarded as a potential consideration for future structural optimization rather than an exclusion criterion, particularly because drug‐likeness rules are heuristic guidelines, and several approved drugs, especially natural products, deviate from these conventional physicochemical boundaries [62, 63].

### 2.9 ADMET Evaluation of Phytochemicals of SNP‐Mixture and Co‐Crystallized Reference Ligands

The ADMET profiles of the investigated phytochemicals, with emphasis on QR‐PME, are presented in Table S2. These ADMET endpoints were estimated with the Web‐based pkCSM platform (https://biosig.lab.uq.edu.au/pkcsm/). Detailed predicted ADMET profiles are provided in Table S2.

### 2.10 Ligand‐Based Target Prediction of Quercetin Pentamethyl Ether (QR‐PME) Using SwissTargetPrediction

**FIGURE 7:**
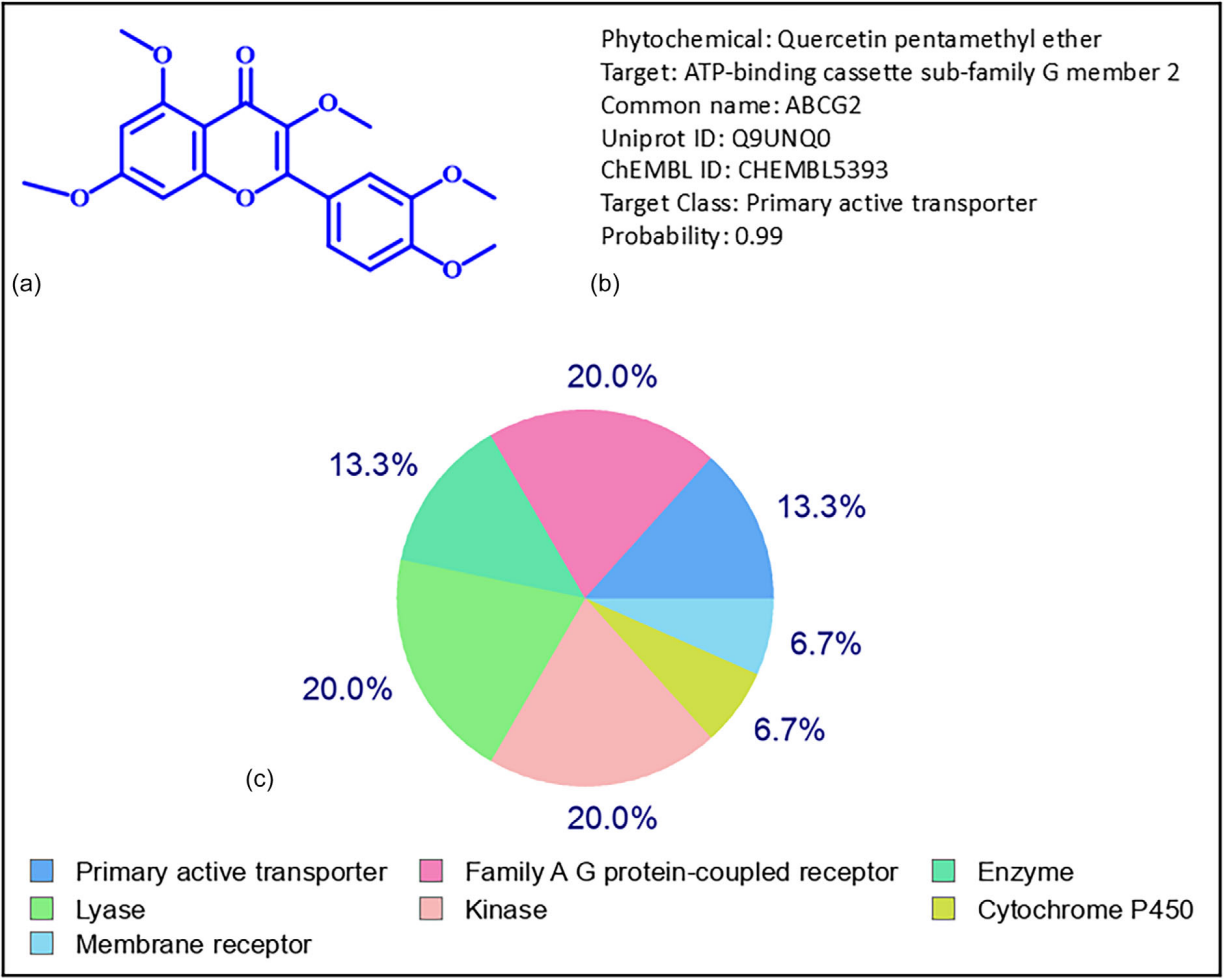
SwissTargetPrediction‐based target profiling of quercetin pentamethyl ether (QR‐PME). (a) Two‐dimensional chemical structure of QR‐PME. (b) SwissTargetPrediction output highlighting ABCG2 as the top‐ranked target, classified as a primary active transporter with a predicted interaction probability of 0.99. (c) Pie chart summarizing the relative proportions (%) of all predicted QR‐PME targets grouped by target class, illustrating the multi‐target intracellular interaction profile of QR‐PME and the prominent contribution of the primary active transporter class that includes ABCG2.

In the SwissTargetPrediction analysis, QR‐PME, a fully O‐methylated derivative of quercetin, was predicted to bind to ATP‐binding cassette transporter G2 (ABCG2) as the top‐ranked human target (Figure 7a–c).

Despite major clinical advances in the treatment of type 2 diabetes mellitus (T2DM) and AD, broadly effective, low‐toxicity, disease‐modifying therapies remain elusive [64], partly because these disorders are molecularly interconnected through convergent networks involving insulin/IGF dysregulation [65], chronic inflammation, oxidative stress [66], and impaired protein quality control (proteostasis failure) [67]. In this study, we applied an integrated structure‐based discovery workflow comprising molecular docking, three independent 100‐ns MD simulations per protein–ligand complex, and MM‐GBSA binding free energy estimates, complemented by physicochemical and drug‐likeness profiling, ADMET screening, and ligand‐based target prediction. Because docking and MM‐GBSA estimate how well a molecule may fit and be stabilized in a binding pocket under defined computational settings, these results were used to prioritize candidates for subsequent experimental evaluation of their mechanisms, selectivity, and biological efficacy. The major phytochemicals of the SNP mixture were therefore screened against AChE, BACE1, DPP4, glucokinase, GSK3β, and TACE—to identify putative target–ligand interaction profiles and generate experimentally testable hypotheses relevant to the intertwined molecular pathways of T2DM and AD.

Among the investigated phytochemicals, QR‐PME emerged as the primary computational hit based on the integrated docking results (Table 2), structural behavior across the triplicate MD trajectories (Figures 5D–F, 6D–F, J–L), and MM‐GBSA estimates obtained using the same protocol as for the reference ligands (Table 3). Its binding free energies toward GSK3β and TACE were notably within a comparable range to those of BRD0705 and hydantoin, differing by less than 1 kcal/mol (Table 3), whereas its predicted interaction strength with DPP4 was less favorable than that of vildagliptin in three independent 100‐ns MD simulations. These findings support an experimentally testable polypharmacological hypothesis centered on the QR‐PME rather than demonstrating simultaneous target modulation, which is particularly relevant because the T2DM–AD axis involves interconnected metabolic, inflammatory, amyloidogenic, and tau‐associated pathways that may not be adequately addressed through a single‐target strategy [68, 69].

To the best of our knowledge, direct molecular recognition of quercetin pentamethyl ether (QR‐PME) by GSK3β has not previously been investigated through an integrated molecular docking and replicated MD simulation approach. GSK3β represents a mechanistically relevant link between T2DM and AD: impaired insulin–PI3K–AKT signaling may release inhibitory control over GSK3β, contributing to defective glucose metabolism, whereas excessive cerebral GSK3β activity promotes tau hyperphosphorylation, neuroinflammation, synaptic dysfunction, and cognitive decline [70, 71, 72]. Indeed, recent findings in ApoE4‐expressing T2DM mice showed increased GSK3β activity and aggravated AD‐like pathology, while pharmacological GSK3β inhibition attenuated these alterations [72]. In this context, QR‐PME showed favorable docking and formed structurally stable GSK3β complexes across three independent 100‐ns simulations, with closely distributed RMSD (0.248–0.271 nm), RMSF (0.093–0.117 nm), and *R*_g_ (2.150–2.181 nm) values (Table 2 and Figure 6D–F). Its mean MM‐GBSA binding free energy (−30.07 ± 0.06 kcal/mol) closely matched that of BRD0705 (−30.05 ± 0.07 kcal/mol), with a difference of 0.02 kcal/mol (Table 3). Despite a low mean H‐bond count (0.41 ± 0.003), binding of QR‐PME was predominantly supported by favorable van der Waals contributions and recurring interactions with Val67 and Leu184, with additional contributions from Phe64, Thr134, and Arg137 (Figure S5 and Table S1). Previous studies reported that pentamethylquercetin modulates AKT–GSK3β signaling or GSK3β phosphorylation in cellular systems but did not establish direct GSK3β binding [73, 74]. Thus, the present results provide a previously unexplored structural basis for QR‐PME–GSK3β recognition and support its prioritization for direct enzyme inhibition, binding kinetics, and cellular validation.

A recent network pharmacology and molecular docking study identified TACE (ADAM17) as a therapeutically relevant target in AD and reported that quercetin, the parent scaffold of QR‐PME, interacted favorably with TACE, with a docking score of −8.21 kcal/mol, consistent with the previously reported anti‐inflammatory activity of quercetin in LPS‐challenged BV‐2 microglial cells [75]. Extending these findings, the present docking study, three independent 100‐ns MD simulations, and MM‐GBSA calculations showed that the more lipophilic, O‐methylated derivative, QR‐PME, maintained stable interactions within the TACE catalytic pocket (Tables 2 and 3; Figure 6J–L). Its mean MM‐GBSA binding free energy (−34.24 ± 0.07 kcal/mol) was closely comparable to that of hydantoin (−34.08 ± 0.06 kcal/mol), with QR‐PME displaying a marginally more negative MM‐GBSA estimate (~0.16 kcal/mol) (Table 3). Despite its modest mean H‐bond count (0.62 ± 0.003), the QR‐PME–TACE interaction was predominantly supported by favorable van der Waals contributions (Table S1), consistent with a binding mode largely governed by hydrophobic and shape complementarity effects.

At the molecular level, Aβ oligomers can disrupt membrane dynamics and/or activate receptor‐driven signaling cascades such as TNFR1/ROS‐mediated pathways, thereby increasing the expression and/or cell‐surface availability of transmembrane TNF (tmTNF; pro‐TNF, 26 kDa) [76]. The tmTNF substrate is then recognized by TACE, the principal membrane “sheddase,” whose catalytic site mediates ectodomain shedding to generate soluble, bioactive sTNF homotrimers. Amplified sTNF–TNFR1 signaling can phosphorylate JNK (c‐Jun N‐terminal kinase) and activate it via the MAPK axis, triggering serine phosphorylation of IRS‐1/2 that exerts an inhibitory effect; this, in turn, downregulates PI3K/Akt signaling and promotes neuronal insulin/IGF‐1 resistance, thereby establishing a key inflammatory–metabolic bridge in AD pathogenesis [77]. Thus, QR‐PME's predicted stability within the TACE active site may provide a mechanistic basis that this ligand–target interaction could be relevant to TNF‐α shedding and downstream inflammatory–metabolic signaling; however, whether QR‐PME can modulate TACE activity, sTNF–TNFR1 signaling, JNK‐mediated IRS phosphorylation, or insulin/IGF signaling requires direct experimental validation.

SwissTargetPrediction ranked ABCG2 as the top‐predicted human target for QR‐PME (Figure 7). As an exploratory analysis, we docked QR‐PME into the central ligand‐binding cavity of human ABCG2 using the MZ82‐bound cryo‐EM structure (PDB ID: 8QCM). QR‐PME adopted a feasible binding pose with an energetically favorable docking score of −7.92 kcal/mol, compared with −9.95 kcal/mol for the co‐crystallized inhibitor MZ82, and formed predominantly hydrophobic contacts with Val401, Leu405, Phe439, Val546, and Met549 (Figure S10). Although these findings provide preliminary structural support for a possible QR‐PME–ABCG2 interaction, docking alone may not establish the binding affinity, transport, inhibition, or activation. A potential interaction could be relevant to AD as ABCG2 is expressed at the blood–brain barrier and in hepatobiliary tissues, where it contributes to xenobiotic removal and amyloid‐β efflux [78]. Consistent with this role, ABCG2, together with ABCG4, has been shown to mediate the efflux of Aβ_1–40_ across the murine blood–brain barrier, thereby limiting its cerebral accumulation [78]. Moreover, quercetin has been shown to stimulate wild‐type ABCG2 ATPase activity at sub‐micromolar concentrations [79]. Thus, QR‐PME, as a closely related quercetin derivative, warrants experimental evaluation for possible ABCG2 interaction; however, its functional directionality (activation vs. inhibition), transport relevance at the blood–brain barrier, and potential effect on amyloid‐β clearance remain entirely open experimental questions.

## 3 Conclusion

In this computational study, structure‐based molecular modeling, physicochemical and ADMET profiling, and ligand‐based target prediction were integrated to prioritize SNP‐mixture phytochemicals against enzymes mechanistically linking metabolic dysfunction, inflammation, and neurodegeneration along the T2DM–AD axis. MD simulations were conducted as three independent 100‐ns trajectories for each receptor–ligand system, with structural stability and energetic convergence assessed through RMSD, RMSF, Rg, and potential energy profiles. MM‐GBSA calculations were subsequently performed over the final 10 ns of each trajectory. Within this framework, QR‐PME emerged as the primary computational hit, displaying stable interaction patterns with GSK3β and TACE and mean MM‐GBSA binding free energies closely comparable to those of the respective reference inhibitors, BRD0705 and hydantoin. These findings support a testable polypharmacological hypothesis in which QR‐PME may interact with targets associated with insulin/tau signaling and TNF‐mediated inflammatory pathways. Its favorable oral drug‐likeness and predicted intestinal absorption further support its prioritization, although its predicted CYP inhibition profile and low blood–brain barrier permeability remain important developability considerations. Importantly, these findings should be interpreted within the methodological boundaries of in silico modeling. Docking scores, MD‐derived stability metrics, and MM‐GBSA estimates enable rational prioritization but do not directly predict potency, selectivity, or in vivo efficacy. The present findings should therefore be regarded as mechanistically grounded hypotheses requiring experimental validation. Priority studies should include direct GSK3β and TACE binding and enzyme inhibition assays, followed by cellular evaluation of tau phosphorylation, TNF‐α shedding, JNK/IRS signaling, and insulin responsiveness. The predicted favorable docking interactions between QR‐PME and ABCG2 should likewise be examined experimentally in models relevant to amyloid‐β transport and clearance. A rational structural optimization strategy of QR‐PME should preserve the methoxy‐substituted flavonoid surface responsible for favorable van der Waals and hydrophobic complementarity while identifying solvent‐exposed methoxy groups that are dispensable for GSK3β and TACE recognition. Selective modification of such positions—through deuteration of metabolically vulnerable O‐methyl groups or replacement with less O‐dealkylation‐prone bioisosteres—could improve microsomal stability and reduce CYP‐mediated interaction liability without substantially compromising permeability or target affinity. These modifications should be guided by metabolite identification studies, CYP phenotyping, and structure–activity analyses performed in parallel with blood–brain barrier and target engagement measurements. Collectively, this study identifies QR‐PME as the principal candidate for experimental evaluation and provides a rational framework for its development as a potential multi‐target modulator of the metabolic–inflammatory–tau network connecting T2DM and AD.

## 4 Experimental Section

### 4.1 Retrieval and Preparation of Phytochemicals

In this computational study, 7‐hydroxycadalene (7HC), euparvic acid (EA), lepidimoic acid (LEA), linolenic acid (LIA), mangiferin (MNG), quercetin pentamethyl ether (QR‐PME), and xanthomicrol (XNM) were retrieved as structure data files (SDF) from PubChem (https://pubchem.ncbi.nlm.nih.gov/) and subsequently used for in silico evaluation against enzymes implicated in the molecular pathogenesis of AD and type 2 diabetes mellitus. Following visual structure checking for ligand integrity, the predominant protonation form of each ligand under physiological conditions (pH 7.4) was predicted using the MolGpKa Web Server (https://xundrug.cn/molgpka/silicopka; see the next section). All phytochemicals, along with co‐crystallized inhibitors/activators retained as reference compounds, were energy‐minimized in Avogadro [80] using the MMFF94 force field and a conjugate gradient minimizer until the energy gradient converged to 1 × 10^−7^.

### 4.2 In Silico pK_a_ Prediction and Protonation State Assignment of Ligands

All ligands investigated in this study—7HC, EA, LEA, LIA, MNG, QR‐PME, and XNM, as presented in Figure 1—as well as the co‐crystallized/reference inhibitors or activators used for comparative docking and MD analyses were first submitted as canonical SMILES strings to the MolGpKa web server (https://xundrug.cn/molgpka/silicopka) [81]. MolGpKa utilizes a graph‐convolutional neural network (GCNN) trained on an extensive experimental and computed pK_a_ database, providing rapid and highly accurate (mean absolute error ≈0.5 pK_a_ units) predictions of microscopic ionization constants (pK_a_). The resulting pK_a_ values were employed to determine the predominant protonation pattern of each ligand under physiological conditions (pH 7.4). Thus, both phytochemicals and reference ligands were treated using the same protonation‐state assignment workflow prior to in silico analyses. For each ionizable acidic or basic center, the dominant protomeric state at pH 7.4 was assigned using the Henderson–Hasselbalch relationships HA ⇌ A^−^ + H^+^ for acidic groups and B + H^+^ ⇌ BH^+^ for basic groups. Acidic centers were considered predominantly deprotonated when pH exceeded the predicted pK_a_, whereas basic centers were considered predominantly protonated when pH was below the predicted pK_a_. Accordingly, 7HC and QR‐PME were retained as neutral protomers. EA, LEA, and LIA were prepared as mono‐anionic carboxylate forms, reflecting dominant deprotonation of their carboxyl groups at a pH of 7.4. XNM was prepared as a mono‐anionic 3‐phenolate protomer, while MNG was prepared as a tri‐anionic phenolate protomer. For the co‐crystallized/reference ligands, the predicted pK_a_ profiles indicated that the inhibitors of AChE, BACE1, GSK3β, and TACE, as well as the glucokinase activator, predominantly exist in their neutral form at pH 7.4, whereas the DPP4 inhibitor vildagliptin was assigned as a mono‐cationic protomer due to protonation of its basic secondary amine center (Table S3).

These assigned protomeric forms were subsequently carried forward into all docking calculations, MD simulations, and in silico ADME‐Tox (ADMET) evaluations. Where pK_a_ analysis predicted an ionized dominant state, the required protonation/deprotonation was carried out—and the appropriate formal charge assigned—using the **Chemistry → Charge** tool in BIOVIA Discovery Studio Visualizer v16; ligands whose prevalent form was neutral were, however, left unmodified. This ensured that the phytochemicals and the co‐crystallized/reference ligands were compared under consistent protonation conditions. Accurate pK_a_ estimation at this early stage is critical since the charge form of a drug candidate may markedly affect its binding energy against the target(s), membrane permeability, metabolic stability, and overall pharmacokinetic profile.

### 4.3 Preparation of Protein Structures

The protein receptors used comprised six X‐ray crystal structures obtained from the RCSB Protein Data Bank (https://www.rcsb.org/) in PDB format: acetylcholinesterase (AChE; **7AIS**, **1.75 Å**), β‐secretase 1 (BACE1; **4X7I**, **1.77 Å**), dipeptidyl peptidase‐4 (DPP4; **6B1E**, **1.77 Å**), glucokinase (**6E0I**, **1.90 Å**), glycogen synthase kinase‐3β (GSK3β; **7SXJ**, **1.85 Å**), and TNF‐α converting enzyme (TACE; **3L0V**, **1.75 Å**). Additionally, the human ABCG2 (**8QCM**, **2.39 Å**) structure in complex with MZ82 and the 5D3 Fab was retrieved and prepared using the same receptor preparation workflow for comparative redocking of MZ82 and exploratory docking of QR‐PME. Subsequently, crystallographic water molecules, buffer ions, and redundant protein subunits were removed prior to molecular docking using Discovery Studio Visualizer [82]. Missing heavy atoms were rebuilt with Swiss‐PdbViewer v4.1 [83], after which gaps in the polypeptide chains were filled by loop modeling in Neurosnap PDBFixer (https://neurosnap.ai/service/PDBFixer). Since PDBFixer resets residue numbering starting from “1,” the modeled coordinates were realigned with the numbering scheme of the original PDB entry using the “Renumber Residue Numbers In Pdb File” web utility (https://www.canoz.com/sdh/renumberpdbchain.pl). Each model‐derived protein structure was subsequently protonated at physiological pH 7.4 with VegaZZ v3.2.3 [84].

### 4.4 Molecular Docking

In the present investigation, AutoDock Vina v1.2.7 was employed to characterize the binding modes of seven phytochemicals (7HC, EA, LEA, LIA, MNG, QR‐PME, and XNM) toward six therapeutically relevant enzymes—acetylcholinesterase (AChE), β‐secretase 1 (BACE1), dipeptidyl‐peptidase 4 (DPP4), glucokinase, glycogen‐synthase‐kinase‐3β (GSK3β), and tumor‐necrosis‐factor‐α‐converting enzyme (TACE) [85]. Nonpolar hydrogen atoms were merged, whereas all polar hydrogens on receptors and ligands were preserved with AutoDockTools 1.5.7. Receptors were assigned Kollman charges, and ligands were assigned Gasteiger charges. The molecular docking followed a semiflexible protocol: each ligand torsion was allowed to undergo full rotation, while protein residue side chains were held rigid. The catalytically relevant active sites of each protein receptor were delineated using the coordinates of the bound co‐crystallized ligands (inhibitors or activators) identified in the corresponding X‐ray structures. For docking, the grid box was centered on the native ligand position and dimensioned to fully cover the catalytic cavity: AChE, 20 × 20 × 20 Å (*x*: 85.48 Å, *y*: –55.38 Å, *z*: 54.13 Å); BACE1, 13 × 13 × 13 Å (*x*: 25.26 Å, *y*: 54.51 Å, *z*: 88.06 Å); DPP4, 13 × 13 × 13 Å (*x*: 38.89 Å, *y*: 50.97 Å, *z*: 36.62 Å); glucokinase, 20 × 20 × 20 Å (*x*: 8.59 Å, *y*: 1.18 Å, *z*: –18.98 Å); GSK3β, 12 × 12 × 12 Å (*x*: 12.92 Å, *y*: –8.86 Å, *z*: 33.30 Å); and TACE, 14 × 14 × 14 Å (*x*: 6.85 Å, *y*: 9.73 Å, *z*: 25.01 Å); and ABCG2, 24 × 24 × 24 Å (*x*: 123.08 Å, *y*: 129.48 Å, *z*: 134.71 Å). Protein residue charges were verified using AutoDockTools “**Check Residue Charge**” tool, which flags any residue whose net charge is nonintegral and evenly redistributes the excess or deficit before PDBQT export. Following this step, all receptors and ligands—including the co‐crystallized inhibitors and activators—were saved in the PDBQT format. Binding energies (docking scores; Δ*G* in kcal/mol) derived from redocking the native ligand–protein complexes were adopted as reference controls for comparative evaluation of the phytochemical docking results. The docking protocol was further validated by calculating the all‐atom root‐mean‐square deviation (RMSD) between each redocked native ligand pose and its corresponding crystallographic conformation. The resulting redocking RMSD values are presented in Table S5. For each docking, the Vina exhaustiveness was fixed at 64, and 20 independent runs were performed per ligand. AutoDock Vina 1.2.7 grouped the predicted binding modes into geometric clusters, after which the poses were ordered by their docking scores. The lowest energy (most negative Δ*G*) pose within this set was taken as the representative best‐ranked conformation. For each receptor, the selected poses of 7HC, EA, LEA, LIA, MNG, QR‐PME, and XNM were subsequently visually evaluated, rendered, and prepared in Discovery Studio Visualizer [82].

### 4.5 Post‐Docking MM‐GBSA Binding Free Energy Calculations

Reliable quantification of ligand–receptor binding free energies is a key component of structure‐based hit identification. Accordingly, we estimated MM‐GBSA (Molecular Mechanics/Generalized Born Surface Area) binding free energies for the top‐ranked docking conformations of the major phytochemicals using Uni‐GBSA (‐‐https://github.com/dptech‐corp/Uni‐GBSA). [86]. Calculations employed an internal dielectric of 1, an external dielectric of 78.5, 150 mM ionic strength, and the Onufriev–Bashford–Case GB‐OBC2 model [87]; all remaining parameters followed Uni‐GBSA defaults. This protocol yielded binding free energy estimates for each ligand (7HC, EA, LEA, LIA, MNG, QR‐PME, XNM, and positive controls) in complex with their targets (DPP4, glucokinase, AChE, BACE1, GSK3β, and TACE), enabling a quantitative comparison of interaction strengths that complements the docking scores.

### 4.6 Molecular Dynamics Simulations of Major Phytochemicals

Starting from the top‐ranked docked receptor–phytochemical poses, we performed all‐atom MD simulations as follows: coordinates of receptors were exported as PDB files using Discovery Studio Visualizer [82] and prepared with standard GROMACS tools [88], using the AMBER03 force field [89]. Ligands were processed in Avogadro [80], hydrogens were added, MOL2 files were generated, and the ACPYPE program [90] was used to produce GROMACS topologies with GAFF2 parameters [91] and Gasteiger partial charges [92]. Protein and ligand topologies were merged to build each complex, which was embedded in a cubic periodic box and solvated with SPC water [93]; systems were ion‐balanced with Na^+^/Cl^−^ to approximate physiological ionic strength. Nonbonded interactions were treated using a 1.2 nm van der Waals cutoff, while long‐range electrostatics were computed with particle‐mesh Ewald (PME) [94]. Each system was minimized by steepest descent, followed by 100 ps of restrained equilibration (protein and ligand; 1000 kJ mol^−1^ nm^−2^) to relax the solvent and ions. Production simulations were propagated for 100 ns under NPT conditions (300 K, 1 atm) using a Berendsen thermostat [93] and an isotropic Parrinello–Rahman barostat [95]. A 2 fs time step was applied, and LINCS constraints were used to fix bonds involving hydrogen atoms [96]. The resulting trajectories from the three independent replicas were processed using standard GROMACS tools to evaluate the structural and energetic behavior of each complex through protein Cα root–mean‐square deviation (RMSD), Cα root‐mean‐square fluctuation (RMSF), radius of gyration (Rg), ligand–protein H‐bond numbers, and potential energy profiles. Binding free‐energy estimates were computed as described below. Finally, trajectory snapshots were visually examined in PyMOL [58]. To ensure methodological consistency, all phytochemical and reference ligand complexes underwent identical parameterization, solvation, ionization, equilibration, production MD, and MM‐GBSA protocols. Production MD simulations were performed as three independently initiated 100‐ns trajectories per complex to assess structural stability, replicate‐to‐replicate variability, and the reproducibility of binding energy estimates.

### 4.7 Post‐MD MM‐GBSA Binding Free Energy Calculations

The average interaction strengths between the principal phytochemicals and their protein targets during the MD simulations were quantified via MM‐GBSA binding free energy analysis [97, 98, 99]. For each system, including positive controls, the analysis was performed separately for the three independent trajectories using 501 configurations extracted at 20‐ps intervals from the equilibrated final 10 ns of each 100‐ns simulation. Calculations employed the GROMACS‐compatible gmx_MMPBSA package [100, 101] using a physiological salt concentration (0.154 M), internal and external dielectric constants of 1 and 78.5, the Onufriev–Bashford–Case GB‐OBC2 implicit‐solvent model [87], a 1.4 Å solvent probe radius, and all other default parameters. Because conformational entropy was not included, the reported MM‐GBSA values should be interpreted as relative, enthalpy‐dominated estimates rather than absolute binding free energies. Potential energy profiles were additionally obtained over each complete 100‐ns trajectory to assess energetic stabilization, identify possible systematic drift, and evaluate consistency among the independent replicas. Using the same package and final‐10‐ns configurations, per‐residue energy decomposition was also performed to identify the principal residues contributing to protein–ligand binding.

### 4.8 Drug‐Likeness Assessment

In SBDD, evaluating the drug‐likeness attributes of candidate compounds is essential for minimizing undesirable biological effects. Drug‐likeness refers to the qualitative appraisal of a molecule's suitability as an orally administered therapeutic, inferred from its predicted pharmacokinetic behavior. This approach aims to filter out hit compounds with largely incompatible molecular properties through the routine screening of chemical compound libraries, seeking an “acceptable pharmacokinetic profile” [102]. The drug‐likeness profiles of the tested compounds, together with the co‐crystallized reference ligands (inhibitors/activators), were assessed via the web‐based SwissADME platform (http://www.swissadme.ch/index.php) [56, 102]. For each ligand, canonical SMILES strings were submitted, and SwissADME summarized oral drug‐likeness using Lipinski's Rule of Five (Ro5) compliance and the predicted bioavailability score (Table 4).

### 4.9 ADMET Prediction

An SBDD hit is expected to show not only favorable binding affinity but also acceptable pharmacokinetic behavior and safety liabilities, collectively denoted as ADMET [103]. Accordingly, ADMET endpoints for the ligands, together with the co‐crystallized reference inhibitors/activators, were computationally predicted using the pkCSM web server (https://biosig.lab.uq.edu.au/pkcsm/) [104, 105]. Detailed predicted ADMET profiles of the SNP‐mixture phytochemicals are provided in Table S2.

### 4.10 Ligand‐Based Target Prediction Using SwissTargetPrediction

The single‐drug/single‐target paradigm in drug discovery exhibits certain limitations in terms of safety and efficacy, as highlighted by Chandran et al. [106]. Consequently, exploring potential off‐target effects of hit or lead compounds and uncovering additional therapeutic activities requires a target‐prediction strategy in which small‐molecule–protein interactions are inferred by ligand‐based comparison of query structures with known ligands of multiple protein targets, thereby elucidating molecular mechanisms, rationalizing favorable or unfavorable side effects, and supporting drug repurposing. In this study, putative human protein targets and the compound–target interaction network for the identified phytochemical hit(s) were predicted by selecting *Homo sapiens* as the target organism and querying the SwissTargetPrediction web server [55] https://www.swisstargetprediction.ch/.

## Author Contributions

**Jean Philippe Djientcheu Tientcheu:** conceptualization (lead), methodology (supporting), validation (supporting), writing – review & editing (supporting). **Erman Salih İstifli:** conceptualization (lead), methodology (lead), investigation (lead), formal analysis (lead), software (lead), validation (lead), supervision (lead), writing – original draft (lead), writing – review & editing (lead).

## Funding

This study was supported by the Third World Academy of Sciences (TWAS) (Grant no: 3240338198).

## Conflicts of Interest

The authors declare no conflicts of interest.

## Supporting information

Supplementary Material

## Data Availability

The data that support the findings of this study are available from the corresponding author upon reasonable demand.
