# Supplementary material for: Integrative In Silico Profiling of Major Phytochemicals From a Sclerocarya birrea–Nauclea latifolia–Piper longum Mixture: Docking, Molecular Dynamics, ADMET, and Ligand‐Based Target Prediction on Shared Pathways in Type 2 Diabetes Mellitus and Alzheimer's Disease
Source: ChemistryOpen. 2026 Sep 15;15(9):e70299. doi: 10.1002/open.70299 (PMC13576934; doi:10.1002/open.70299)
Supplement: Supplementary file 1 [file OPEN-15-e70299-s001.pdf]

Supporting Information

for

Integrative *In Silico* Profiling of Major Phytochemicals from a *Sclerocarya birrea–Nauclea latifolia–Piper longum* Mixture: Docking, Molecular Dynamics, ADMET, and Ligand-Based Target Prediction on Shared Pathways in Type 2 Diabetes Mellitus and Alzheimer's Disease

Jean Philippe Djientcheu Tientcheu[1] | Erman Salih İstifli[2]

[1]Department of Animal Biology and Physiology, University of Yaound´e I, P.O. Box 812, Yaound´e, Cameroon

[2]Cukurova University, Faculty of Science and Literature, Department of Biology, Adana, Türkiye

Correspondence: Erman S. ISTIFLI (esistifli@cu.edu.tr)

CONTENTS

Fig. S1. Per-residue MM-GBSA energy decomposition heatmaps and bar plots for AChE complexes with the reference inhibitor (A–B) and LIA (C–D) ....

Fig. S2. Per-residue MM-GBSA energy decomposition heatmaps and bar plots for BACE1 complexes with aminothiazine (A–B) and MNG (C–D). ....

Fig. S3. Per-residue MM-GBSA energy decomposition heatmaps and bar plots for DPP4 complexes with vildagliptin (A–B) and QR-PME (C–D) ....

Fig. S4. Per-residue MM-GBSA energy decomposition heatmaps and bar plots for glucokinase (GK) complexes with compound 72 (activator; A–B) and linolenic acid (LIA; C–D) ....

Fig. S5. Per-residue MM-GBSA energy decomposition heatmaps and bar plots for glycogen synthase kinase-3β (GSK3β) complexes with BRD0705 (inhibitor; A–B) and quercetin pentamethyl ether (QR-PME; C–D)........

Fig. S6. Per-residue MM-GBSA energy decomposition heatmaps and bar plots for tumor necrosis factor-α-converting enzyme (TACE/ADAM17) complexes with hydantoin (inhibitor; A–B) and quercetin pentamethyl ether (QR-PME; C–D)........

Fig. S7. Potential energy profiles of the AChE–reference inhibitor, AChE–LIA, BACE1–reference inhibitor, and BACE1–MNG systems obtained from three independent 100-ns molecular dynamics simulations (Rep 1–3)........

Fig. S8. Potential energy profiles of the DPP4–reference inhibitor, DPP4–QR-PME, GK–reference activator, and GK–LIA systems obtained from three independent 100-ns molecular dynamics simulations (Rep 1–3) ....

Fig. S9. Potential energy profiles of the GSK3β–reference inhibitor, GSK3β–QR-PME, TACE–reference inhibitor, and TACE–QR-PME systems obtained from three independent 100-ns molecular dynamics simulations (Rep 1–3)........

Fig. S10. Molecular docking results of the co-crystallized inhibitor and QR-PME with human ATP-binding cassette transporter G2 (ABCG2; PDB ID: 8QCM) ....

Table S1. Free energy components and total MM-GBSA binding free energies (kcal mol^{-1}) of the reference inhibitors/activator and major phytochemicals in complex with DPP4, glucokinase, AChE, BACE1, GSK3β, and TACE, calculated from three independent 100-ns molecular dynamics simulations........

Table S2. ADMET evaluation of SNP-mixture phytochemicals and co-crystallized reference ligands........

Table S3. Protonation-state assignments of phytochemicals and co-crystallized/reference ligands at pH 7.4........

Table S4. Potential energy convergence statistics from three independent 100-ns molecular dynamics simulations of DPP4, glucokinase (GK), AChE, BACE1, GSK3β, and TACE complexes with their reference ligands and major phytochemicals ....

Table S5. Redocking validation results ....

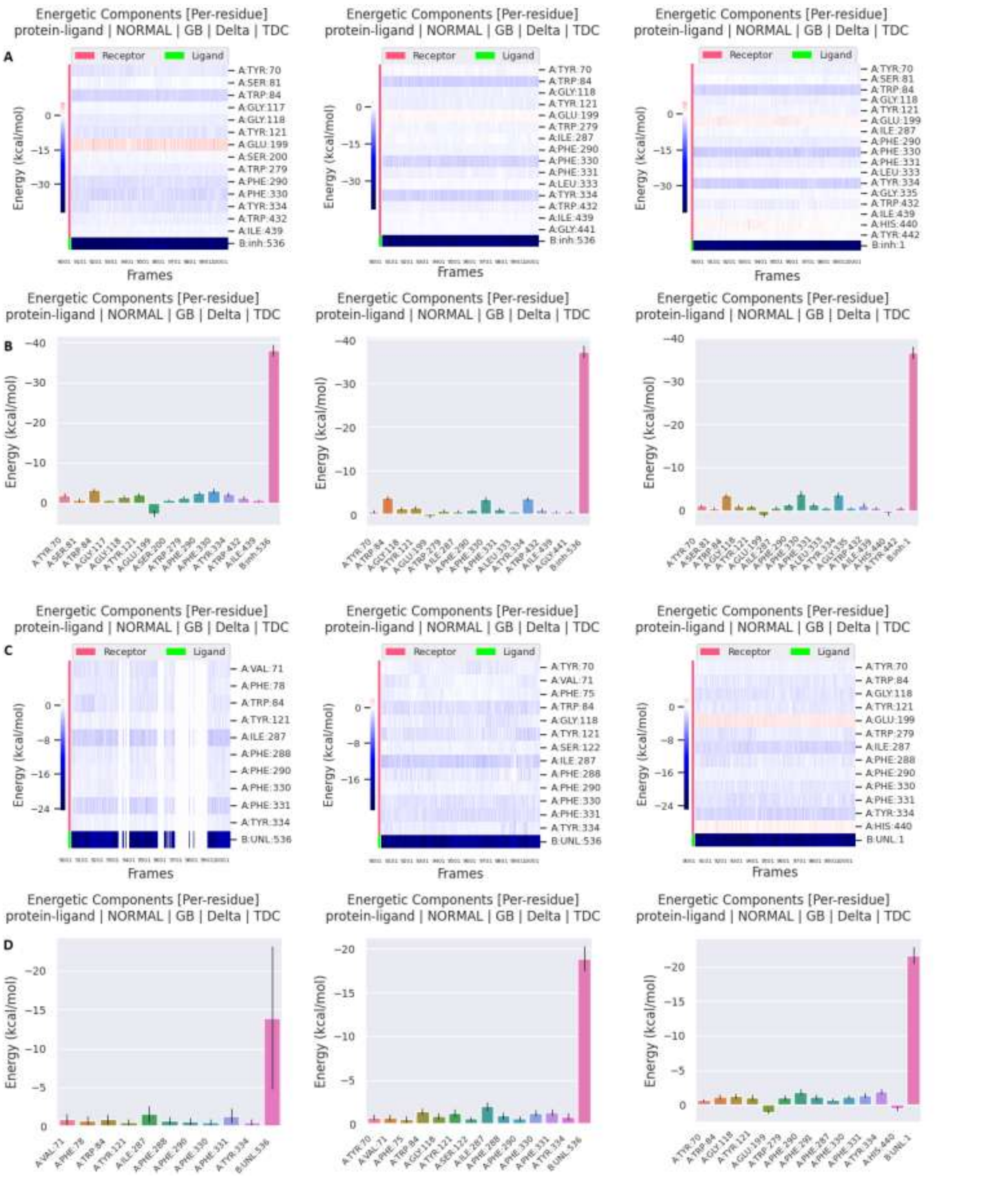

Fig. S1. Per-residue MM-GBSA energy decomposition heatmaps and bar plots for AChE complexes with the reference inhibitor (**A–B**) and LIA (**C–D**). Replicates 1–3 are arranged from left to right. More negative (darker blue) energy contributions indicate residues favorably contributing to protein–ligand binding.

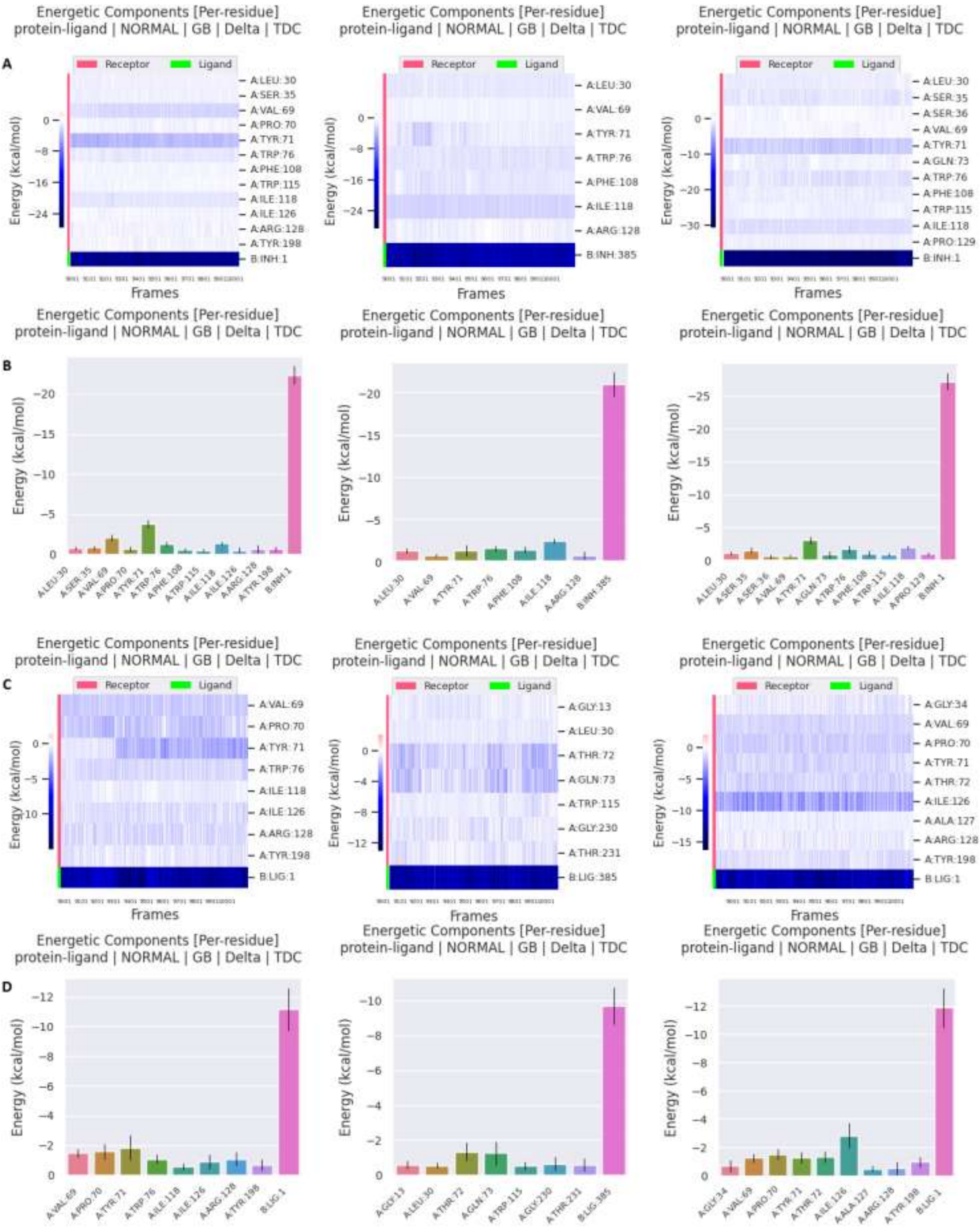

Fig. S2. Per-residue MM-GBSA energy decomposition heatmaps and bar plots for BACE1 complexes with aminothiazine (**A–B**) and MNG (**C–D**). Replicates 1–3 are arranged from left to right. More negative (darker blue) energy contributions indicate residues favorably contributing to protein–ligand binding.

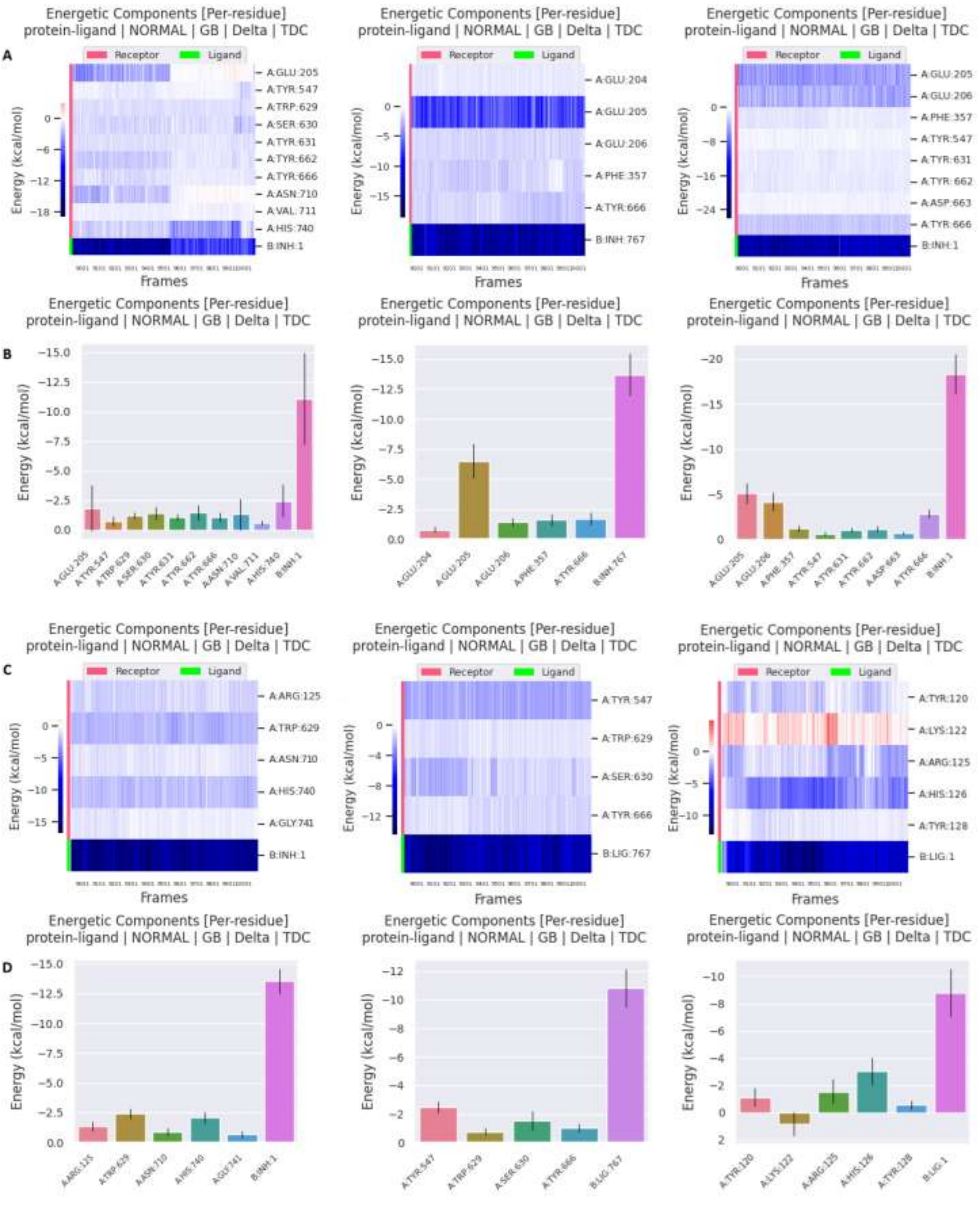

Fig. S3 Per-residue MM-GBSA energy decomposition heatmaps and bar plots for DPP4 complexes with vildagliptin (**A–B**) and QR-PME (**C–D**). Replicates 1–3 are arranged from left to right. More negative (darker blue) energy contributions indicate residues favorably contributing to protein–ligand binding.

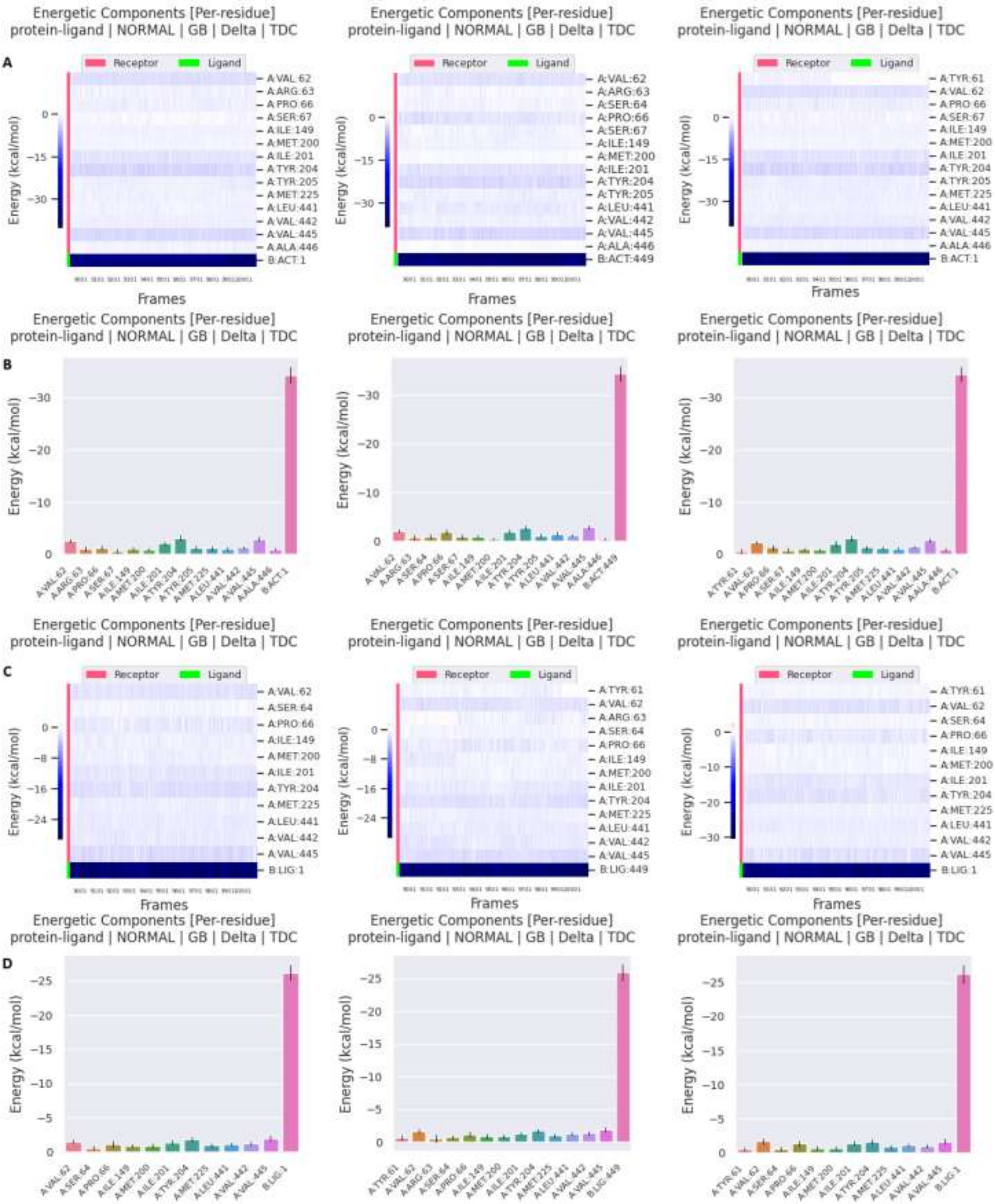

Fig. S4 Per-residue MM-GBSA energy decomposition heatmaps and bar plots for glucokinase (GK) complexes with compound 72 (activator; **A–B**) and linolenic acid (LIA; **C–D**). Replicates 1–3 are arranged from left to right. More negative (darker blue) energy contributions indicate residues favorably contributing to protein–ligand binding.

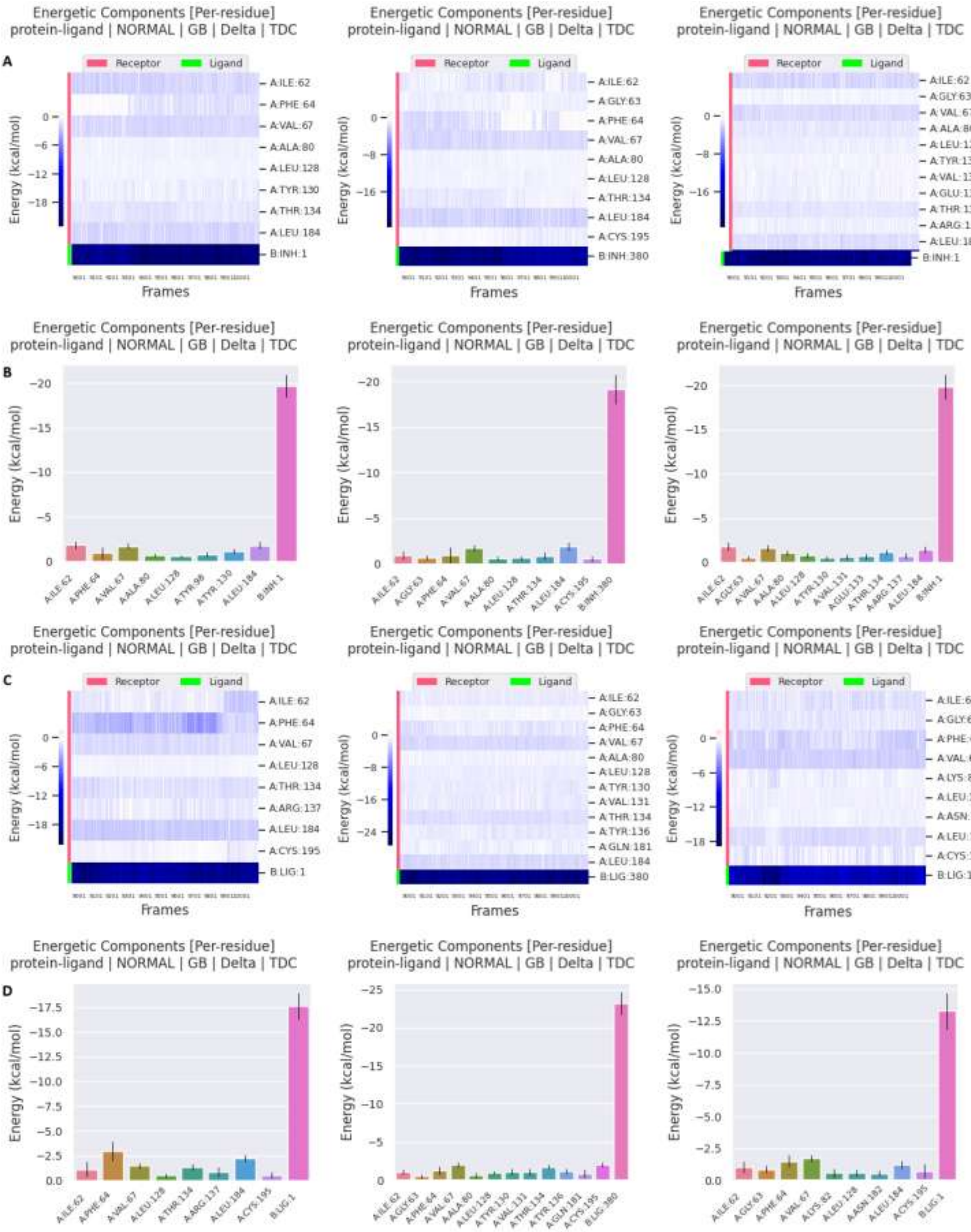

Fig. S5 Per-residue MM-GBSA energy decomposition heatmaps and bar plots for glycogen synthase kinase-3 beta (GSK3β) complexes with BRD0705 (inhibitor; **A–B**) and quercetin pentamethyl ether (QR-PME; **C–D**). Replicates 1–3 are arranged from left to right. More negative (darker blue) energy contributions indicate residues favorably contributing to protein–ligand binding.

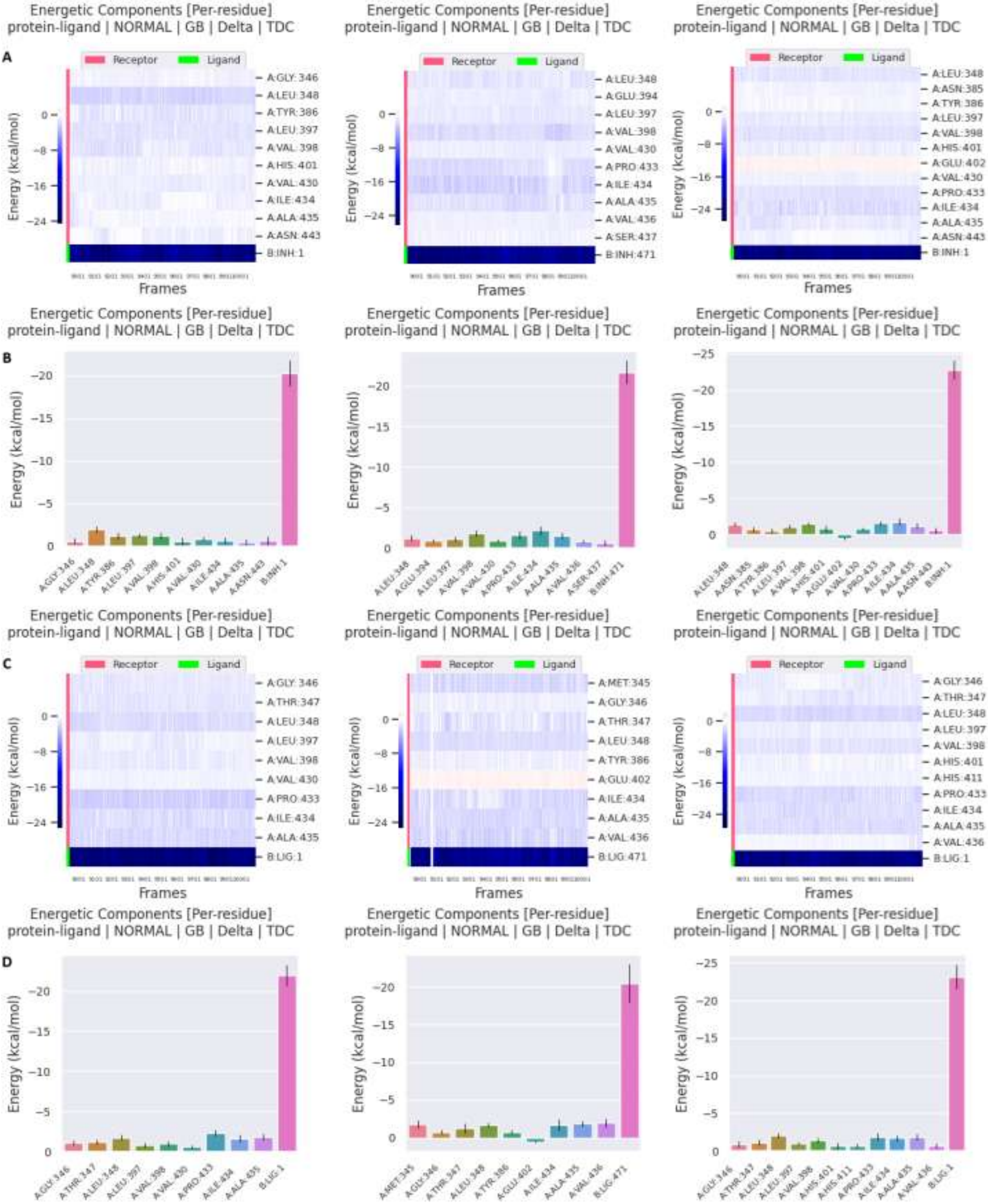

Fig. S6 Per-residue MM-GBSA energy decomposition heatmaps and bar plots for tumor necrosis factor-α-converting enzyme (TACE/ADAM17) complexes with hydantoin (inhibitor; **A–B**) and quercetin pentamethyl ether (QR-PME; **C–D**). Replicates 1–3 are arranged from left to right. More negative (darker blue) energy contributions indicate residues favorably contributing to protein–ligand binding.

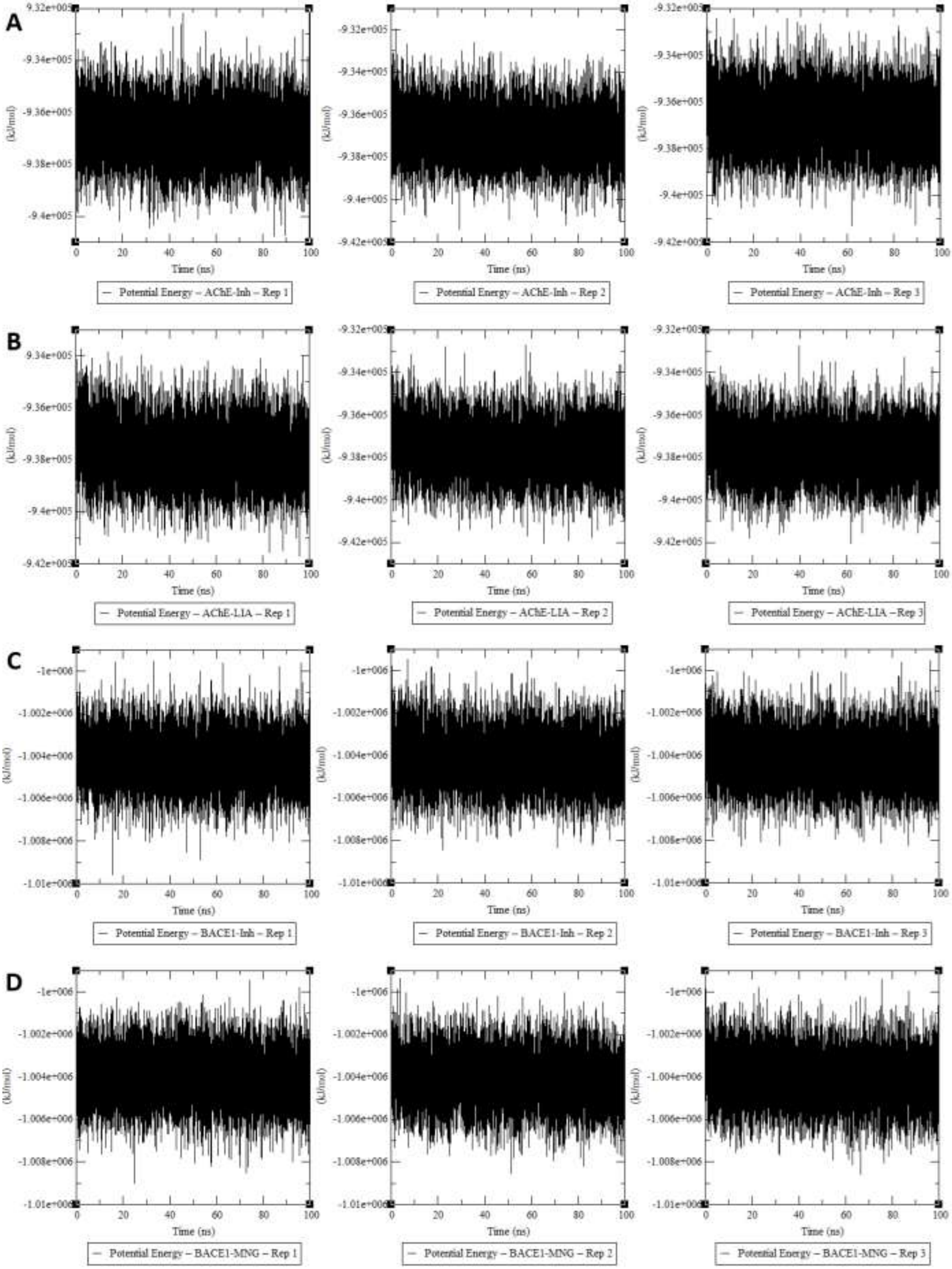

Fig. S7 Time-dependent potential energy profiles of the AChE–reference inhibitor (A), AChE–linolenic acid (LIA; B), BACE1–reference inhibitor (C), and BACE1–mangiferin (MNG; D) systems over three independent 100-ns molecular dynamics simulations. Replicates 1–3 are arranged from left to right. The absence of pronounced long-term drift was consistent with stable energetic behavior over the simulated timescale.

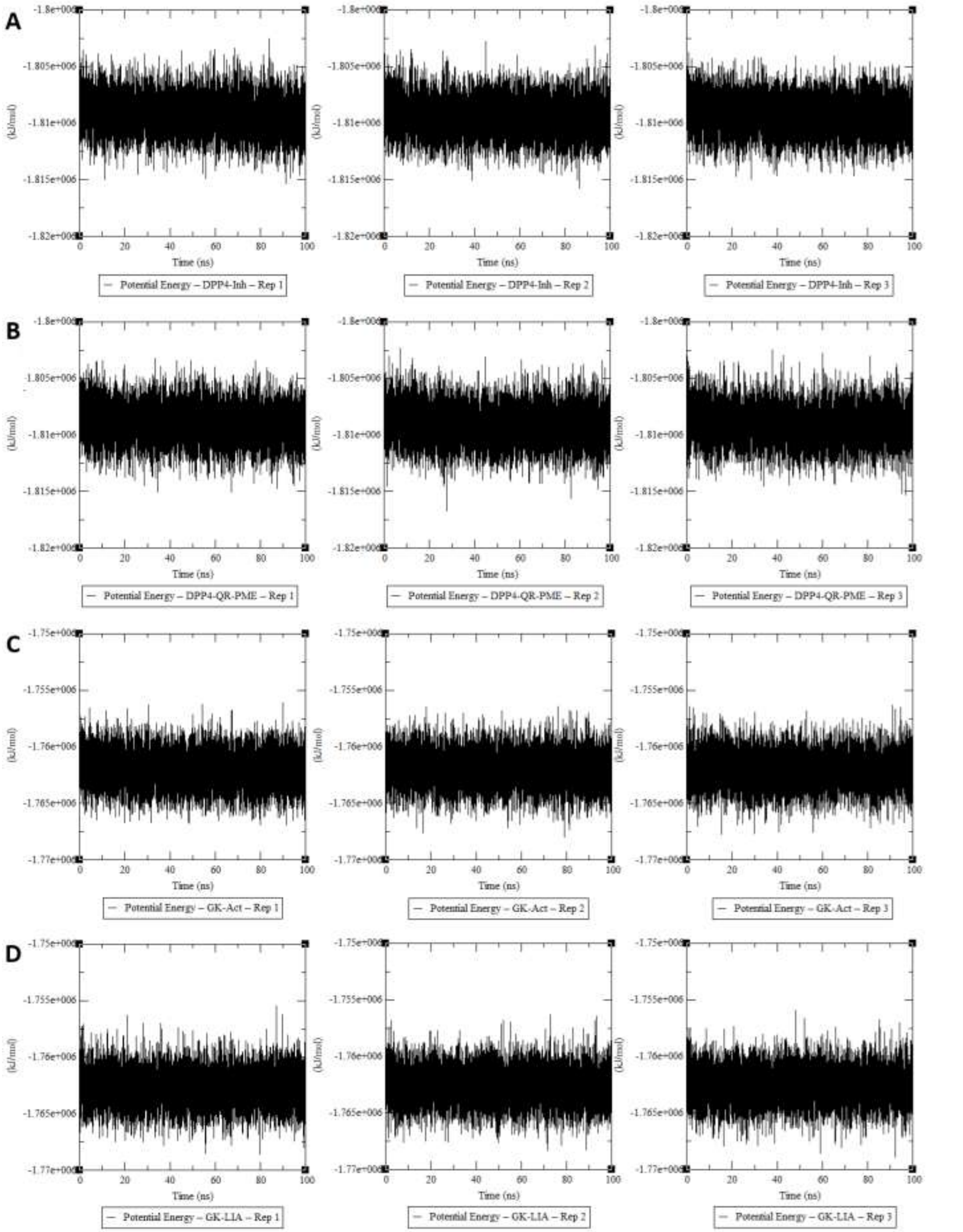

Fig. S8 Time-dependent potential energy profiles of the DPP4–reference inhibitor (A), DPP4–quercetin pentamethyl ether (QR-PME; B), glucokinase (GK)–reference activator (C), and GK–linolenic acid (LIA; D) systems over three independent 100-ns molecular dynamics simulations. Replicates 1–3 are arranged from left to right. The absence of pronounced long-term drift was consistent with stable energetic behavior over the simulated timescale.

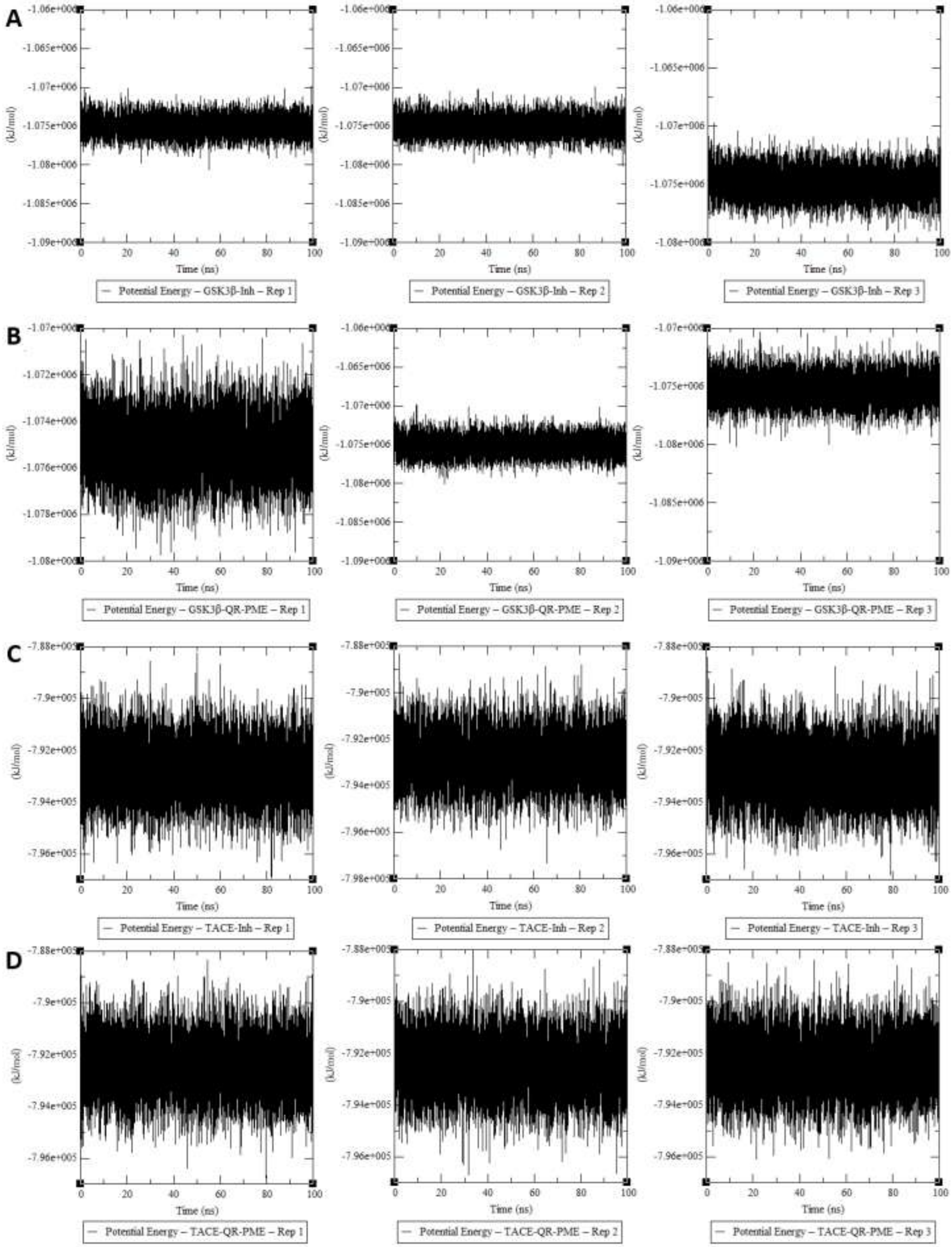

Fig. S9 Time-dependent potential energy profiles of the GSK3β–reference inhibitor (A), GSK3β–quercetin pentamethyl ether (QR-PME; B), TACE–reference inhibitor (C), and TACE–QR-PME (D) systems over three independent 100-ns molecular dynamics simulations. Replicates 1–3 are arranged from left to right. The absence of pronounced long-term drift was consistent with stable energetic behavior over the simulated timescale.

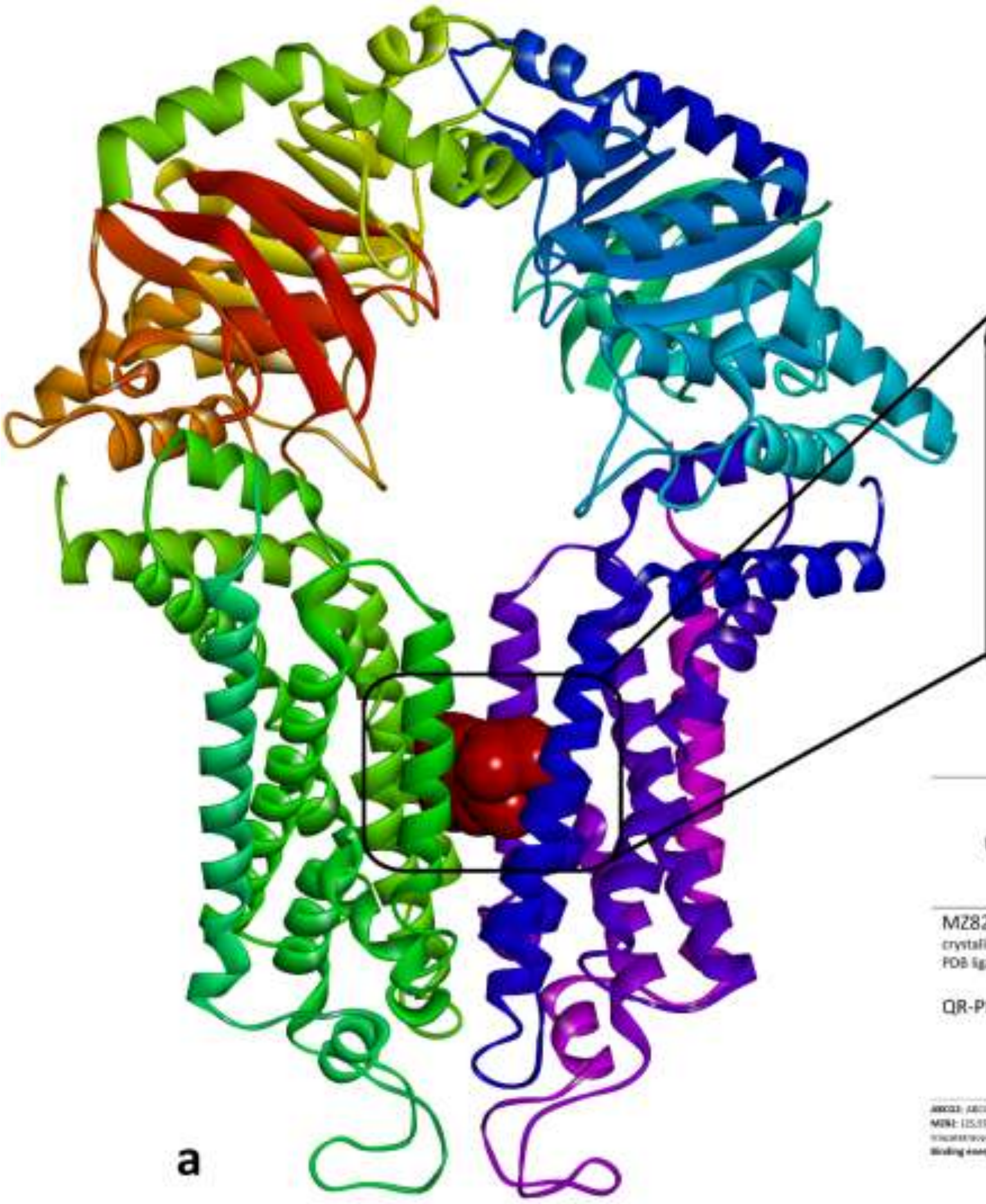

a

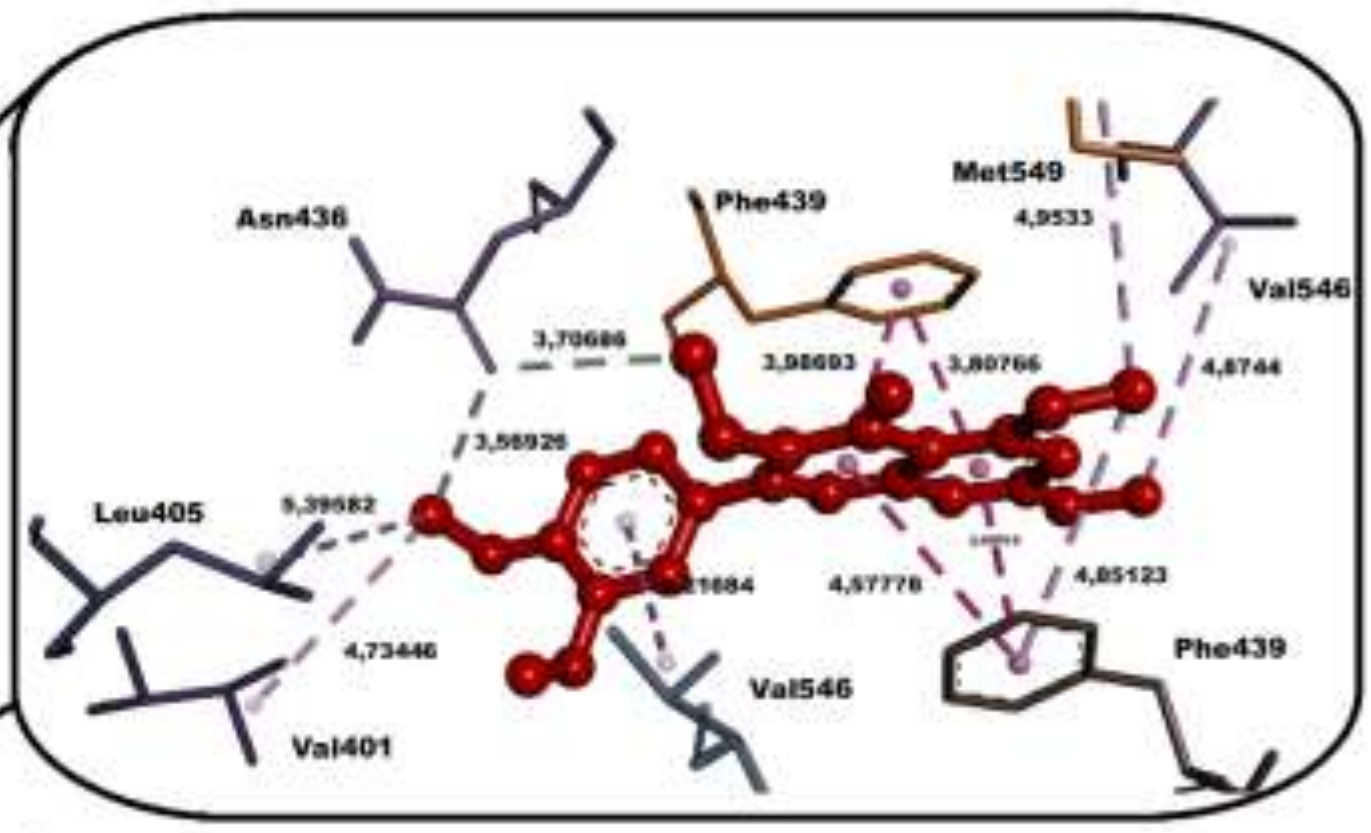

b

Ligand	Protein	Binding energy (docking score) (kcal mol⁻¹)	Molecular interactions	
			H-bonds	Hydrophobic
MZ82* (co-crystallized inhibitor; PDB ligand ID: V0U)	ABCG2*	-9.95	Asn436 (3.57 Å, 3.71 Å)	Val401 (4.73 Å), Leu405 (5.40 Å), Phe439 (3.81 Å, 3.96 Å, 3.99 Å, 4.58 Å, 4.85 Å), Val546 (4.87 Å, 5.22 Å), Met549 (4.95 Å)
QR-PME	ABCG2	-7.92	–	Val401 (4.45 Å), Leu405 (4.55 Å, 5.17 Å), Phe439 (4.15 Å, 4.15 Å, 4.69 Å, 5.54 Å), Pro485 (5.39 Å), Ile543 (3.64 Å, 5.20 Å), Val546 (3.72 Å, 4.99 Å)

Fig. S10 Molecular docking results of the co-crystallized inhibitor and QR-PME with human ATP-binding cassette transporter G2 (ABCG2).

Table S1 Free energy components and total MM-GBSA binding free energies (kcal/mol) of the reference inhibitors/activator and major phytochemicals in complex with DPP4, glucokinase, AChE, BACE1, GSK3β, and TACE, calculated from three independent 100-ns molecular dynamics simulations.

		MM-GBSA Free Energy (kcal/mol)							H-BONDS
**Ligand—Receptor**	R.	$\Delta E_{VDWAALS}$	ΔE_{EEL}	ΔE_{EGB}	ΔE_{ESURF}	ΔE_{GGAS}	ΔE_{GSOLV}	ΔE_{TOTAL}	Mean number
**Vildagliptin (inhibitor)—DPP4**	1	-31.69 ± 0.11	-259.80 ± 1.00	271.21 ± 0.88	-4.17 ± 0.01	-291.49 ± 1.08	267.05 ± 0.87	-24.44 ± 0.24	1.62 ± 0.008
	2	-29.30 ± 0.08	-257.13 ± 0.32	264.63 ± 0.29	-4.23 ± 0.01	-286.43 ± 0.32	260.39 ± 0.29	-26.04 ± 0.08	1.42 ± 0.007
	3	-29.11 ± 0.11	-305.97 ± 0.46	305.32 ± 0.38	-3.80 ± 0.01	-335.08 ± 0.46	301.52 ± 0.38	-33.55 ± 0.14	2.11 ± 0.008
								**-28.01 ± 0.10**	**1.72 ± 0.005**
**QR-PME—DPP4**	1	-25.64 ± 0.06	0.65 ± 0.11	8.29 ± 0.10	-3.00 ± 0.01	-24.98 ± 0.13	5.29 ± 0.10	-19.69 ± 0.06	0.35 ± 0.006
	2	-27.01 ± 0.06	-1.71 ± 0.10	16.47 ± 0.07	-3.71 ± 0.01	-28.71 ± 0.12	12.76 ± 0.07	-15.96 ± 0.08	0.36 ± 0.005
	3	-22.29 ± 0.15	-4.94 ± 0.25	17.45 ± 0.26	-2.67 ± 0.02	-27.23 ± 0.32	14.77 ± 0.25	-12.45 ± 0.14	0.33 ± 0.005
								**-16.03 ± 0.06**	**0.35 ± 0.003**
**Compound 72 (activator)—Glucokinase**	1	-65.99 ± 0.12	4.33 ± 0.15	12.67 ± 0.14	-7.71 ± 0.01	-61.66 ± 0.19	4.96 ± 0.14	-56.71 ± 0.13	1.52 ± 0.006
	2	-64.78 ± 0.09	-3.88 ± 0.14	19.59 ± 0.13	-7.70 ± 0.01	-68.66 ± 0.17	11.89 ± 0.13	-56.77 ± 0.10	0.84 ± 0.008
	3	-65.96 ± 0.12	1.19 ± 0.11	15.79 ± 0.10	-7.68 ± 0.01	-64.76 ± 0.16	8.11 ± 0.10	-56.66 ± 0.13	1.33 ± 0.006
								**-56.71 ± 0.07**	**1.23 ± 0.004**
**Linolenic acid—Glucokinase**	1	-44.83 ± 0.10	-6.87 ± 0.10	16.34 ± 0.08	-6.59 ± 0.01	-51.70 ± 0.13	9.75 ± 0.08	-41.95 ± 0.10	0.48 ± 0.005
	2	-44.16 ± 0.08	-7.46 ± 0.07	16.04 ± 0.05	-6.50 ± 0.01	-51.62 ± 0.09	9.53 ± 0.05	-42.09 ± 0.08	0.42 ± 0.005
	3	-44.42 ± 0.10	-8.19 ± 0.15	17.57 ± 0.14	-6.82 ± 0.01	-52.61 ± 0.18	10.75 ± 0.14	-41.86 ± 0.11	0.25 ± 0.004
								**-41.97 ± 0.06**	**0.38 ± 0.003**
**Compound 5b (co-crystallized inhibitor)—AChE**	1	-76.55 ± 0.09	-7.36 ± 0.07	31.30 ± 0.07	-8.47 ± 0.01	-83.91 ± 0.11	22.84 ± 0.07	-61.08 ± 0.09	0.27 ± 0.005
	2	-76.58 ± 0.09	-0.93 ± 0.07	26.64 ± 0.07	-8.47 ± 0.01	-77.51 ± 0.13	18.17 ± 0.07	-59.34 ± 0.09	0.04 ± 0.002
	3	-74.16 ± 0.13	-8.21 ± 0.11	32.09 ± 0.12	-8.07 ± 0.01	-82.37 ± 0.17	24.02 ± 0.11	-58.35 ± 0.14	0.07 ± 0.003
								**-59.59 ± 0.06**	**0.13 ± 0.002**
**Linolenic acid—AChE**	1	-30.16 ± 0.62	-10.71 ± 0.23	20.82 ± 0.43	-4.48 ± 0.09	-40.87 ± 0.84	16.35 ± 0.34	-24.52 ± 0.51	0.78 ± 0.006
	2	-41.70 ± 0.08	-16.51 ± 0.11	30.60 ± 0.10	-5.98 ± 0.01	-58.20 ± 0.14	24.62 ± 0.10	-33.58 ± 0.09	1.14 ± 0.006
	3	-48.15 ± 0.10	-16.45 ± 0.13	34.63 ± 0.12	-7.12 ± 0.01	-64.60 ± 0.17	27.51 ± 0.12	-37.09 ± 0.11	1.24 ± 0.006
								**-31.73 ± 0.18**	**1.05 ± 0.003**
**Aminothiazine (inhibitor)—BACE1**	1	-45.30 ± 0.07	-6.66 ± 0.09	22.38 ± 0.08	-6.00 ± 0.01	-51.96 ± 0.12	16.39 ± 0.08	-35.58 ± 0.07	0.84 ± 0.007
	2	-39.90 ± 0.08	-15.68 ± 0.09	28.23 ± 0.08	-5.84 ± 0.01	-55.58 ± 0.14	22.39 ± 0.08	-33.19 ± 0.09	0.58 ± 0.006
	3	-53.12 ± 0.13	-11.82 ± 0.11	26.58 ± 0.12	-6.68 ± 0.01	-64.94 ± 0.18	19.90 ± 0.11	-45.04 ± 0.12	0.90 ± 0.007
								**-37.94 ± 0.06**	**0.77 ± 0.004**
**Mangiferin—BACE1**	1	-31.88 ± 0.14	-10.41 ± 0.17	26.73 ± 0.16	-4.63 ± 0.02	-42.29 ± 0.22	22.10 ± 0.15	-20.19 ± 0.12	1.14 ± 0.009
	2	-29.88 ± 0.06	-3.90 ± 0.12	24.25 ± 0.13	-4.02 ± 0.01	-33.78 ± 0.13	20.23 ± 0.12	-13.55 ± 0.08	1.30 ± 0.011
	3	-33.52 ± 0.11	-12.45 ± 0.17	27.99 ± 0.14	-4.85 ± 0.01	-45.97 ± 0.20	23.15 ± 0.14	-22.82 ± 0.12	0.97 ± 0.009
								**-18.85 ± 0.06**	**1.14 ± 0.006**
**BRD0705 (inhibitor)—GSK3β**	1	-37.02 ± 0.11	-4.98 ± 0.05	16.57 ± 0.05	-4.66 ± 0.01	-42.01 ± 0.12	11.91 ± 0.05	-30.10 ± 0.12	0.48 ± 0.007
	2	-36.43 ± 0.09	-2.40 ± 0.06	14.89 ± 0.05	-4.79 ± 0.01	-38.83 ± 0.13	10.10 ± 0.05	-28.73 ± 0.10	0.25 ± 0.006
	3	-37.08 ± 0.12	-2.40 ± 0.08	12.59 ± 0.08	-4.44 ± 0.01	-39.49 ± 0.14	8.15 ± 0.08	-31.33 ± 0.12	0.72 ± 0.006
								**-30.05 ± 0.07**	**0.48 ± 0.004**
**Quercetin-PME—GSK3β**	1	-40.45 ± 0.12	-0.81 ± 0.19	17.27 ± 0.18	-5.65 ± 0.01	-41.26 ± 0.24	11.62 ± 0.17	-29.65 ± 0.11	0.20 ± 0.004
	2	-48.44 ± 0.08	-3.15 ± 0.10	18.19 ± 0.08	-6.40 ± 0.01	-51.59 ± 0.12	11.79 ± 0.08	-39.80 ± 0.09	0.60 ± 0.006
	3	-31.55 ± 0.09	-3.31 ± 0.21	18.49 ± 0.17	-4.39 ± 0.01	-34.86 ± 0.23	14.10 ± 0.17	-20.76 ± 0.10	0.43 ± 0.006
								**-30.07 ± 0.06**	**0.41 ± 0.003**

		MM-GBSA Free Energy (kcal/mol)							H-BONDS
**Ligand—Receptor**	R.	$\Delta E_{VDWAALS}$	ΔE_{EEL}	ΔE_{EGB}	ΔE_{ESURF}	ΔE_{GGAS}	ΔE_{GSOLV}	ΔE_{TOTAL}	Mean number
**Hydantoin (inhibitor)—TACE**	1	-38.19 ± 0.10	-11.79 ± 0.22	23.07 ± 0.19	-4.78 ± 0.01	-49.98 ± 0.25	18.28 ± 0.19	-31.70 ± 0.12	1.34 ± 0.010
	2	-41.08 ± 0.08	-13.03 ± 0.07	23.48 ± 0.06	-4.47 ± 0.01	-54.12 ± 0.10	19.01 ± 0.06	-35.11 ± 0.08	1.67 ± 0.011
	3	-42.07 ± 0.09	-12.32 ± 0.11	23.80 ± 0.08	-4.84 ± 0.01	-54.39 ± 0.14	18.96 ± 0.08	-35.43 ± 0.11	1.83 ± 0.011
								**-34.08 ± 0.06**	**1.61 ± 0.006**
**Quercetin-PME—TACE**	1	-44.54 ± 0.10	-9.25 ± 0.12	24.31 ± 0.12	-5.04 ± 0.01	-53.80 ± 0.16	19.27 ± 0.12	-34.53 ± 0.11	0.34 ± 0.005
	2	-37.81 ± 0.14	-11.00 ± 0.12	22.10 ± 0.12	-4.47 ± 0.01	-48.81 ± 0.20	17.63 ± 0.11	-31.18 ± 0.13	0.82 ± 0.006
	3	-45.87 ± 0.13	-4.64 ± 0.15	18.93 ± 0.12	-5.42 ± 0.01	-50.52 ± 0.18	13.51 ± 0.12	-37.01 ± 0.13	0.72 ± 0.006
								**-34.24 ± 0.07**	**0.62 ± 0.003**

Values for individual replicates are reported as mean ± SEM from the corresponding MM-GBSA or hydrogen-bond trajectory analysis.

For each complex, the bold summary row reports the arithmetic mean across three independent 100-ns simulations; uncertainty was calculated as the propagated SEM: $\sqrt{(SEM_1^2 + SEM_2^2 + SEM_3^2)}/3$.

ΔG_GAS = ΔE_VDWAALS + ΔE_EL; ΔG_SOLV = ΔE_GB + ΔE_SURF; ΔG_TOTAL = ΔG_GAS + ΔG_SOLV. All energy terms are expressed in kcal/mol.

DPP4, dipeptidyl peptidase-4; GK, glucokinase; AChE, acetylcholinesterase; BACE1, β-secretase 1; GSK3β, glycogen synthase kinase 3β; TACE, TNF-α-converting enzyme.

Compound 5b is the co-crystallized AChE inhibitor in PDB structure 7AIS (PDB ligand identifier: 8UH).

Table S2 ADMET evaluation of phytochemicals of SNP-mixture and co-crystallized reference ligands

ADMET analysis	Compounds						
	**7HC**	**LIA**	**EA**	**LEA**	**MNG**	**QR-PME**	**XNM**
**Absorption**							
$CaCO_2$ permeability (log Papp in 10^{-6} cm/s)	1.551	1.577	0.627	-0.496	-0.926	1.208	0.515
GI absorption (human)	High	High	Moderate	Low	Low	High	Moderate
P-gp substrate	Yes	No	Yes	Yes	Yes	No	Yes
P-gp inhibitor	No	No	No	No	No	Yes	No
**Distribution**							
VDss (human) (log L/kg)	0.770	-0.617	-0.724	0.058	1.364	-0.220	0.110
Fraction unbound (human)	0	0.056	0.391	0.782	0.289	0.191	0.014
BBB permeability	High	Moderate	Low	Low	Very low	Low	High
**Metabolism**							
CYP2D6 substrate	No	No	No	No	No	No	No
CYP3A4 substrate	Yes	Yes	No	No	No	Yes	Yes
CYP1A2 inhibitor	Yes	Yes	No	No	No	Yes	Yes
CYP2C19 inhibitor	Yes	No	No	No	No	Yes	Yes
CYP2C9 inhibitor	No	No	No	No	No	Yes	Yes
CYP2D6 inhibitor	No	No	No	No	No	No	No
CYP3A4 inhibitor	No	Yes	No	No	No	Yes	Yes
**Excretion**							
Total Clearance (log mL/min/kg)	0.161	1.991	0.491	1.338	0.347	0.817	0.657
Renal OCT2 substrate	No	No	No	No	No	No	No
**Toxicity**							
AMES toxicity (Mutagenicity)	No	No	No	No	No	No	No
hERG inhibitor	No	No	No	No	No	No	No
Oral Rat Acute Toxicity (LD_{50}) (mol/kg)	2.144	1.441	2.383	1.867	2.396	2.365	2.341
Hepatotoxicity	No	Yes	No	No	No	No	No
T. pyriformis toxicity (log μg/L)	1.331	0.722	0.281	0.285	0.285	0.364	0.337
Minnow toxicity (log mM)	-0.748	-1.183	1.940	6.801	5.898	0.242	1.647

OCT2 = Organic Cation Transporter 2; BBB = Blood-brain barrier, P-gp = P-glycoprotein, VDss = steady-state volume of distribution, 7HC = 7-Hydroxycadalene, LIA = Linolenic Acid, EA = Euparvic Acid, LEA = Lepidimoic Acid, MNG = Mangiferin, QR-PME = Quercetin Pentamethyl Ether, XNM = Xanthomicrol

Absorption

The ADMET profiles of the investigated SNP-mixture phytochemicals are summarized in Table S2, with particular emphasis on QR-PME as the primary hit. QR-PME showed a favorable predicted absorption profile, combining high Caco-2 permeability (log Papp = 1.208) with high gastrointestinal absorption. It was not predicted to be a P-glycoprotein (P-gp) substrate, suggesting limited susceptibility to P-gp-mediated efflux; however, its predicted P-gp inhibitory activity indicates a potential transporter-mediated drug–drug interaction liability that should be experimentally evaluated.

Distribution

QR-PME exhibited a relatively low predicted steady-state volume of distribution (VDss = −0.220 log L/kg), with a human unbound fraction of 0.191. Its predicted low blood–brain barrier permeability suggests limited passive penetration into the central nervous system. Although this may constrain direct exposure to central targets, BBB permeability remains a model-dependent prediction that requires confirmation using experimental permeability and pharmacokinetic studies.

Metabolism

QR-PME was predicted to be a CYP3A4 substrate and to inhibit CYP1A2, CYP2C19, CYP2C9, and CYP3A4, while showing no predicted substrate or inhibitory activity toward CYP2D6. This multi-CYP inhibition profile represents the principal metabolic liability identified for QR-PME and suggests a potential risk of CYP-mediated drug–drug interactions. Targeted scaffold optimization and experimental CYP inhibition assays will therefore be important to determine whether this liability can be reduced while preserving its favorable absorption and target-interaction profiles.

Excretion

QR-PME showed moderate predicted total clearance (0.817 log mL/min/kg) and was not identified as a renal organic cation transporter 2 (OCT2) substrate. The latter finding suggests a low likelihood of OCT2-mediated renal transport interactions, although definitive conclusions regarding renal elimination and systemic half-life will require experimental pharmacokinetic assessment.

Toxicity

The predicted toxicity profile of QR-PME was generally favorable: it was negative for Ames mutagenicity, hERG inhibition, and hepatotoxicity and showed a predicted oral rat acute toxicity value of 2.365 mol/kg. The *T. pyriformis* and minnow toxicity values were 0.364 log μg/L and 0.242 log mM, respectively. Because these ecological toxicity endpoints are derived from non-human models, they have limited direct relevance to human safety and should be regarded primarily as supportive screening indicators. Overall, QR-PME combines favorable predicted intestinal absorption and an absence of major mutagenic, cardiotoxic, or hepatotoxic alerts, although its low predicted BBB permeability and potential P-gp/CYP inhibition liabilities warrant targeted experimental validation.

Table S2 (*continued*)

ADMET analysis	Co-crystallized inhibitors/activator					
	**AChE inhibitor[1]**	**BACE1 inhibitor[2]**	**GSK3β inhibitor[3]**	**TACE inhibitor[4]**	**DPP4 inhibitor[5]**	**Glucokinase activator**
**Absorption**						
$CaCO_2$ permeability (log Papp in 10^{-6} cm/s)	0.439	1.442	1.343	0.836	0.591	1.089
GI absorption (human)	Low	High	High	High	High	Low
P-gp substrate	Yes	Yes	Yes	No	No	Yes
P-gp inhibitor	Yes	No	Yes	No	No	Yes
**Distribution**						
VDss (human) (log L/kg)	-0.067	0.447	0.425	-0.084	0.586	0.793
Fraction unbound (human)	0	0.173	0.005	0.377	0.569	0.217
BBB permeability	Low	Low	Moderate	Low	Low	Very low
**Metabolism**						
CYP2D6 substrate	Yes	No	No	No	No	No
CYP3A4 substrate	Yes	Yes	Yes	No	No	Yes
CYP1A2 inhibitor	No	No	Yes	No	No	No
CYP2C19 inhibitor	Yes	No	Yes	No	No	No
CYP2C9 inhibitor	Yes	No	Yes	No	No	No
CYP2D6 inhibitor	No	No	No	No	No	No
CYP3A4 inhibitor	Yes	No	No	No	No	Yes
**Excretion**						
Total Clearance (log mL/min/kg)	-0.056	0.068	0.8	0.624	1.006	0.007
Renal OCT2 substrate	No	No	No	No	No	No
**Toxicity**						
AMES toxicity (Mutagenicity)	No	No	No	No	No	No
hERG inhibitor	Yes (hERG-II)	No	Yes (hERG-II)	No	No	Yes (hERG-II)
Oral Rat Acute Toxicity (LD50) (mol/kg)	2.19	2.576	1.033	1.611	2.674	2.839
Hepatotoxicity	Yes	Yes	Yes	Yes	No	Yes
T. pyriformis toxicity (log ug/L)	0.289	0.346	0.679	0.280	0.268	0.285
Minnow toxicity (log mM)	-2.398	2.460	1.739	2.913	2.893	2.272

OCT2 = Organic Cation Transporter 2; BBB = Blood-brain barrier, P-gp = P-glycoprotein, VDss = steady-state volume of distribution; [1]AChE inhibitor: 6-[(3-Chloro-6,7,10,11-tetrahydro-9-methyl-7,11 methanocycloocta[b]quinolin-12-yl)amino]-N-(4-hydroxy-3-methoxybenzyl)hexanamide; [2]BACE1 inhibitor: Aminothiazine (LY2886721); [3]GSK3β inhibitor: BRD0705 ((4~[S])-4-ethyl-7,7-dimethyl-4=penyl-2,6,8,9-tetrahydropyrazolo[3,4-b]quinoline-5-one); [4]TACE inhibitor: Hydantoin ((5R)-5-[(5-methoxy-3-oxo-1,3-dihydro-2H-indazol-2-yl)methyl]-5-methylimidazolidine-2,4-dione); [5]DPP4 inhibitor: Vildagliptin; Glucokinase activator: Compound 72 (1-[4-[5-[[3-(2-methylpyridin-3-yl)oxy-5-pyridin-2-ylsulfanyl-pyridin-2-yl]amino]-1,2,4-thiadiazol-3-yl]piperidin-1-yl]ethanone)

Table S3 Protonation state assignment of phytochemicals and co-crystallized/reference ligands at pH 7.4.

Ligand	Ligand type	Relevant MolGpKa pK_a / ionization result at pH 7.4	Assigned protonation state used in modelling	Net charge	Equation
7HC (7-hydroxycadalene)	Phytochemical	Phenolic OH pK_a ≈ 10.0; >99.7% neutral at pH 7.4	Neutral phenolic protomer	0	Acidic HH; neutral HA retained
EA (euparvic acid)	Phytochemical	COOH pK_a 4.5; ≈99.9% carboxylate at pH 7.4; phenolic OH groups mostly neutral	Mono-anionic carboxylate with neutral phenolic OH groups	−1	Acidic HH; anionic A^- form selected
LEA (lepidimoic acid)	Phytochemical	COOH pK_a 4.0; ≈99.96% carboxylate at pH 7.4; OH groups essentially neutral	Mono-anionic carboxylate form	−1	Acidic HH; anionic A^- form selected
LIA (α-linolenic acid)	Phytochemical	Terminal COOH pK_a 4.8; ≈99.8% carboxylate at pH 7.4	Mono-anionic carboxylate form	−1	Acidic HH; anionic A^- form selected
MNG (mangiferin)	Phytochemical	Phenolic OH sites with pK_a 6.0, 6.3, and 6.8 predominantly deprotonated at pH 7.4	Tri-anionic phenolate protomer	−3	Acidic HH (phenolic); tri-anionic form selected
QR-PME, (quercetin pentamethyl ether)	Phytochemical	No ionizable group detected by MolGpKa	Neutral non-ionized protomer	0	No ionizable group; neutral form retained
XNM (xanthomicrol)	Phytochemical	Phenolic OH pK_a values 7.3 and 8.3; 3-OH is the major deprotonated site at pH 7.4	Mono-anionic 3-phenolate protomer	−1	Acidic HH (phenolic); mono-anionic form selected
Vildagliptin	Co-crystallized DPP4 inhibitor	Basic secondary amine predicted to be protonated at pH 7.4	Mono-cationic protomer	+1	Basic HH; cationic BH^+ form selected
Compound 72	Co-crystallized glucokinase activator	No dominant ionized form at pH 7.4 after pK_a-based evaluation	Neutral protomer	0	Acid–base HH; neutral form retained
8UH	Co-crystallized AChE inhibitor	No dominant ionized form at pH 7.4 after pK_a-based evaluation	Neutral protomer	0	Acid–base HH; neutral form retained
Aminothiazine	Co-crystallized BACE1 inhibitor	No dominant ionized form at pH 7.4 after pK_a-based evaluation	Neutral protomer	0	Acid–base HH; neutral form retained
BRD0705	Co-crystallized GSK3β inhibitor	No dominant ionized form at pH 7.4 after pK_a-based evaluation	Neutral protomer	0	Acid–base HH; neutral form retained
Hydantoin	Co-crystallized TACE inhibitor	No dominant ionized form at pH 7.4 after pK_a-based evaluation	Neutral protomer	0	Acid–base HH; neutral form retained

HH: Henderson–Hasselbalch equation. For an acidic group ($HA \rightleftharpoons A^- + H^+$), $pH = pK_a + \log_{10}([A^-]/[HA])$; for the conjugate acid of a basic group ($BH^+ \rightleftharpoons B + H^+$), $pH = pK_a(BH^+) + \log_{10}([B]/[BH^+])$.

Table S4. Potential energy (kJ/mol) convergence statistics from three independent 100-ns MD simulations (n=3) of the AChE, BACE1, DPP4, glucokinase (GK), GSK3β, and TACE complexes with their reference ligands and selected phytochemicals

System	Rep 1 mean ± SEM (kJ/mol)	Rep 2 mean ± SEM (kJ/mol)	Rep 3 mean ± SEM (kJ/mol)	Mean across replicas ± SD (kJ/mol)
AChE–inhibitor (8UH)	−936753.50 ± 10.92	−936861.17 ± 12.14	−936760.97 ± 12.37	**−936791.88 ± 60.12**
AChE–LIA	−937700.21 ± 11.21	−937564.07 ± 12.25	−937702.74 ± 12.34	**−937655.68 ± 79.34**
BACE1–inhibitor (aminothiazine)	−1004147.69 ± 12.04	−1004138.10 ± 12.22	−1004142.03 ± 12.14	**−1004142.61 ± 4.82**
BACE1–MNG	−1003994.68 ± 12.02	−1004062.66 ± 11.91	−1004029.27 ± 12.14	**−1004028.87 ± 33.99**
DPP4–vildagliptin	−1809222.39 ± 16.52	−1809436.22 ± 16.50	−1809350.87 ± 16.25	**−1809336.49 ± 107.64**
DPP4–QR-PME	−1809010.36 ± 16.52	−1809144.64 ± 16.60	−1809108.46 ± 16.54	**−1809087.82 ± 69.48**
GK–activator (compound 72)	−1762043.68 ± 15.49	−1761997.09 ± 15.36	−1761987.56 ± 15.33	**−1762009.44 ± 30.03**
GK–LIA	−1762735.87 ± 15.42	−1762625.76 ± 15.65	−1762609.85 ± 15.45	**−1762657.16 ± 68.63**
GSK3β–inhibitor (BRD0705)	−1074942.71 ± 12.42	−1074921.66 ± 12.35	−1074875.90 ± 12.33	**−1074913.43 ± 34.16**
GSK3β–QR-PME	−1075101.61 ± 12.39	−1075158.86 ± 12.44	−1075196.98 ± 12.20	**−1075152.48 ± 48.00**
TACE–hydantoin	−792726.71 ± 10.54	−792821.11 ± 10.54	−792813.33 ± 10.54	**−792787.05 ± 52.40**
TACE–QR-PME	−792449.03 ± 10.51	−792468.04 ± 10.43	−792412.39 ± 10.52	**−792443.15 ± 28.29**

SEM: Standard error of the mean.

SD: Standard deviation.

n = 3: Three independent 100-ns molecular dynamics simulation replicas were run for each protein-ligand system.

Across three independent 100-ns MD replicas, the AChE–8UH, AChE–LIA, BACE1–aminothiazine, BACE1–MNG, DPP4–vildagliptin, DPP4–QR-PME, GK–compound 72, GK–LIA, GSK3β–BRD0705, GSK3β–QR-PME, TACE–hydantoin, and TACE–QR-PME systems exhibited closely distributed mean potential energies, with small trajectory-level SEM values (10.43–16.60 kJ mol^{-1}) relative to the magnitude of the total system energies. The standard deviations among the three replicas ranged from 4.82 to 107.64 kJ mol^{-1}, indicating reasonable — though system-dependent — reproducibility among independently initiated simulations. As total system potential energy is dominated by solvent contributions, this metric primarily reflects overall system stability rather than local binding site behavior; together with the absence of pronounced systematic drift in the corresponding time-dependent potential energy profiles (**Figures S7–S9**), these results are consistent with energetic behavior across replicas.

Table S5. Redocking validation results

Molecule	Chemical structure	RMSD (Å)
AChE inhibitor (Compound 5b)	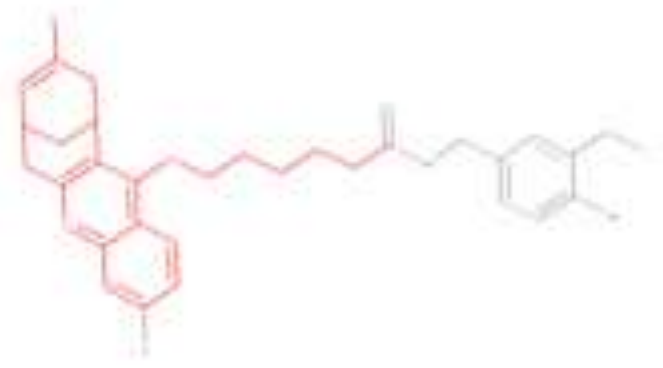	0.71
BACE1 inhibitor (aminothiazine)	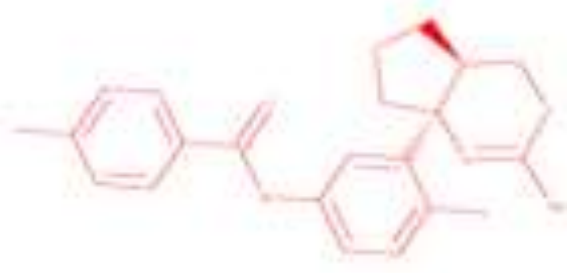	2.97
DPP4 inhibitor (vildagliptin)	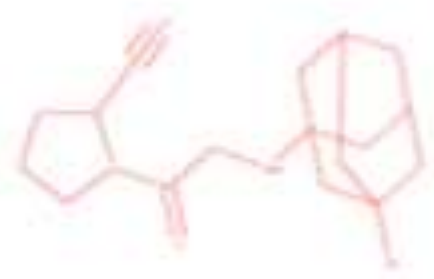	1.55
Glucokinase (GK) activator (Compound 72)	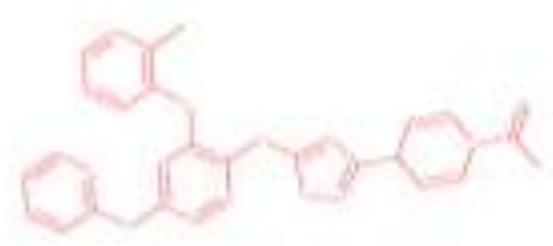	1.28
GSK3β inhibitor (BRD0705)	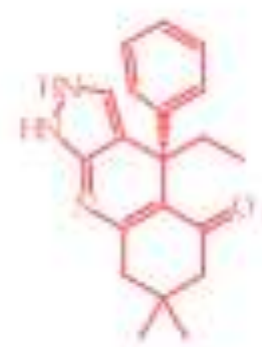	2.08
TACE inhibitor (hydantoin)	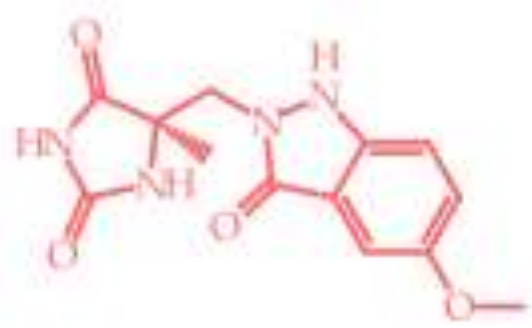	1.87
